# Molecular Diagnostics and Control of Zoonotic Dermatophytosis: First Detection of *Trichophyton indotineae* in a Dog in Africa

**DOI:** 10.3390/ani15172622

**Published:** 2025-09-07

**Authors:** Hend A. Zineldar, Wafaa M. El-Neshwy, Romeo T. Cristina, Nasser Z. Abouzeid, Mohammed I. Eisa, Florin Muselin, Eugenia Dumitrescu, Adel Abdelkhalek, Yasmine H. Tartor

**Affiliations:** 1 Animal Infectious Diseases Department, Faculty of Veterinary Medicine, Zagazig University, Zagazig 44511, Egypt; hadel19@vet.zu.edu.eg (H.A.Z.); wafaaesmail@zu.edu.eg (W.M.E.-N.); nzmohamed@vet.zu.edu.eg (N.Z.A.); mesharaf@vet.zu.edu.eg (M.I.E.); 2 Pharmacology and Toxicology Department, Faculty of Veterinary Medicine, University of Life Sciences “King Mihai I” from Timisoara, Calea Aradului 119, 300645 Timisoara, Romania; romeocristina@usvt.ro (R.T.C.); eugeniadumitrescu@usvt.ro (E.D.); 3 Food Safety, Hygiene and Technology Department, Faculty of Veterinary Medicine, Badr University in Cairo (BUC), Badr 11829, Egypt; adel.abdelkhalek@buc.edu.eg; 4 Microbiology Department, Faculty of Veterinary Medicine, Zagazig University, Zagazig 44511, Egypt

**Keywords:** dermatophytes, disease control, molecular diagnosis, one health, treatment, *Trichophyton indotineae*, zoonosis

## Abstract

Fungal skin infections caused by dermatophytes are of significant veterinary and zoonotic concern. This study investigated the prevalence, risk factors, and treatment outcomes of dermatophytosis in dogs and cats with skin lesions. A total of 140 animals (90 dogs and 50 cats) that presented with skin lesions (alopecia, crusts, pruritus, and scales) were examined. Identification of dermatophyte isolates relied on both phenotypic characteristics and molecular techniques. The molecular approach involved PCR and sequencing of ribosomal DNA regions (ITS1, ITS2, and 5.8S rDNA), complemented by Restriction Fragment Length Polymorphism (RFLP). Treatment regimens included topical clotrimazole, systemic itraconazole, supportive therapy, and a dermatophyte vaccine. Of the 90 dog samples examined, 47 (52.22%) were positive for fungal culture (*p* = 0.65). In cats, 35 samples (70%) were positive (*p* < 0.0001). *Microsporum canis* was the most significant (*p* < 0.0001) prevalent isolate (60%), followed by *Trichophyton mentagrophytes* (20%). This study reported the first isolation of *T. indotineae* in Egypt. Younger animals (<1 year) exhibited a significantly (*p* < 0.05) higher risk of infection compared with adults (>1 year). Indoor/outdoor housing in cats significantly reduced the risk of infection when compared with indoor housing (*p* < 0.0001; odds ratio = 0.05). Combination therapy using itraconazole, clotrimazole, supportive treatment, and a dermatophyte vaccine resulted in the fastest clinical recovery (*p* < 0.0001). These findings underscore the importance of accurate diagnosis and effective treatment strategies for managing dermatophytosis in pets and preventing zoonotic transmission.

## Simple Summary

Dermatophytosis (ringworm) is a zoonotic disease that poses growing risks to both animal and public health. This study provides key insights into dermatophyte species infecting dogs and cats, detection methods, risk factors, and treatment outcomes. *Microsporum canis* was the most predominant cause of infection. Age, breed, housing environment, season, and stress factors were recognized as the primary risk factors for cats. Younger dogs (<1 year), compared with adult dogs (>1 year), exhibited a higher risk of infection. This study provides the first isolation of *Trichophyton indotineae* “*T. mentagrophytes* genotype VIII”, a species typically found in humans, from a dog in Africa. This finding is alarming, as it suggests a potential expansion of the fungus’ host range and highlights its potential as an emerging public health threat. For treatment, results showed that a combination approach using itraconazole, clotrimazole, supportive therapy, and a dermatophyte vaccine was associated with the most efficient recovery. Given the zoonotic nature of *M. canis* and the emerging threat of *T. indotineae*, these findings underscore the critical need for a One Health approach. Accurate diagnosis, effective treatment protocols, and ongoing surveillance are essential to mitigate the risks associated with zoonotic transmission and the emergence of drug-resistant fungi.

## 1. Introduction

Dermatophytosis (ringworm) is a clinically significant fungal skin infection, primarily affecting the keratinized tissues of skin, hair, and claws in dogs and cats [1]. This infection is caused by keratinophilic fungi of the genera *Microsporum, Trichophyton*, and *Nannizzia,* which demonstrate zoophilic, geophilic, or anthropophilic ecological adaptations [2,3]. Dermatophytes were traditionally classified into three main genera: *Trichophyton*, *Microsporum*, and *Epidermophyton*. However, with the discovery of sexual stages and advances in phylogenetic studies, the classification has now been expanded to include six more genera: *Arthroderma*, *Nannizzia*, *Lophophyton*, *Guarromyces*, *Ctenomyces,* and *Paraphyton* [4].

*Trichophyton indotineae* “*T. mentagrophytes* genotype VIII” is an emerging, terbinafine-resistant dermatophyte that belongs to the *T. mentagrophytes/T. interdigitale* complex and has recently been reported in humans globally across the Middle East, Europe, Australia, North America, and Asia [5,6,7,8,9]. Notably, its isolation from felines and canines in India [10] and a stray dog in Iran [11] raises zoonotic transmission concerns.

Dermatophytes transmission occurs primarily through direct contact with an infected host (particularly felines) or indirectly via contaminated fomites [3]. Cats are significant reservoirs of *Microsporum canis*, a highly contagious dermatophyte responsible for zoonotic infections, particularly in pediatric populations [12]. In some rural areas, up to 80% of all human fungal skin infections may be of animal origin [13,14]. The domestication of dogs has increased the zoonotic exposure potential [12].

Although dermatophytosis is a growing concern, research into the associated risk factors in dogs and cats is limited. Factors facilitating mycotic dermatitis include immunosuppression, poor hygiene, and animal and environmental stressors [12,13]. Moreover, traumatic skin lesions and scratching due to flea- and other ectoparasite-related itching facilitate fungal infections [12,14]. Feline and canine dermatophytosis cause polymorphous clinical features that can be reasonably included in the differential diagnosis of all skin diseases in cats and dogs [12]. The pleomorphic presentation of clinical signs, infectious and contagious nature, and zoonosis of dermatophyte infection have made dermatophytosis a condition of veterinary and public health relevance [3].

Animals with skin lesions are more likely to test positive for dermatophytes [3]. While these fungi are more frequently found in animals with skin lesions, they can also be present in unaffected carriers. Indeed, a recent study [15] found a significant difference (*p* = 0.011) in the isolation rate, with 14.3% in dogs and cats with skin lesions compared to 2.6% in those without visible skin lesions.

Accurate and rapid identification of dermatophyte infections is crucial for effective control of disease outbreaks [16]. Molecular techniques, such as polymerase chain reaction (PCR) [17] and restriction fragment length polymorphism (RFLP) analysis using restriction endonucleases such as *Mva*I, enhance diagnostic specificity and sensitivity [18]. Early diagnosis and rapid treatment of dermatophytosis are essential to shorten disease duration and prevent transmission to animals and humans [3].

Topical treatment of superficial dermatophytosis is intended to reduce the infectious, contagious, and zoonotic risks by disinfecting the hair coat and limiting environmental contamination. Oral itraconazole is considered among the most effective systemic antifungal options for both dogs and cats. It has a prolonged half-life and the ability to concentrate in adipose tissue, sebaceous glands, hair, and skin. This pharmacokinetic profile enables pulse therapy, which decreases the treatment cost. Clinical success has been achieved with protocols involving daily administration for one week, followed by alternating weeks of treatment and rest [3,19].

From this perspective, this study aimed to (i) determine the prevalence of different dermatophyte species infecting dogs and cats, (ii) identify associated risk factors, (iii) evaluate diagnostic method concordance, and (iv) assess treatment efficacy using local and/or systemic therapies, supportive treatments, and antifungal vaccines.

## 2. Materials and Methods

### 2.1. Study Population

This study was conducted on 140 animals (90 dogs and 50 cats) presented to veterinary clinics and animal shelters across six Egyptian governorates between May 2022 and May 2023. The study included both owned pets (72 dogs and 50 cats) and sheltered animals (18 dogs), which were examined for clinical signs suggestive of superficial mycoses, including alopecia, erythema, peripheral scaling, crusting, and pruritus. Animals were only included if they exhibited dermatological lesions suspected to be of fungal origin, ensuring that the selected cases were highly indicative of dermatophytosis. Animals were consecutively enrolled based on clinical presentation during the study period and were included after confirming that they fulfilled the inclusion criteria. Animals receiving ongoing antifungal drugs were excluded from the study. Additionally, animals with existing diseases that were adequately controlled by antifungals were also excluded. The sampling period was divided into four seasons: summer (June–August), autumn (September–November), winter (December–February), and spring (March–May) to evaluate seasonal trends in dermatophyte infections. Complete data regarding age, sex, breed, housing environment (indoors/outdoors), hygiene measures, deworming practices, and locality were obtained by interviewing the owners and performing clinical examinations of the animals (Supplementary Table S1).

All animals underwent a detailed dermatological examination. The site and distribution patterns of the lesions were recorded. Wood’s lamp (Burton Medical, Oldsmar, FL, USA) was used as a preliminary screening to detect fluorescence suggestive of *M. canis* infections. To ensure accuracy, Wood’s lamp examination was followed by direct microscopic examination and fungal culture to confirm the presence of dermatophytes. Cases with lesions resembling bacterial pyoderma, autoimmune skin diseases, or allergic dermatitis were excluded based on clinical and diagnostic findings. This multi-step diagnostic approach was applied for all included animals, ensuring that only confirmed cases of superficial mycoses were included in the study, mitigating the potential limitations of Wood’s lamp sensitivity.

Written informed consent was obtained from the owners of the animals involved in the study. The experimental protocol of this study was approved by the Zagazig University Institutional Animal Care and Use Committee (approval number ZU-IACUC/2/F/415/2022). All experiments were performed in accordance with the ARRIVE guidelines and regulations (https://arriveguidelines.org, accessed on 1 February 2022).

### 2.2. Sample Collection

The selected areas for sampling were first gently disinfected using gauze moistened with 70% alcohol. The fur was plucked with forceps, and the skin scales were scraped using a sterilized scalpel blade. Samples were obtained from the periphery of the most recent lesions, identified based on active inflammatory borders characterized by erythema, alopecia, and peripheral scaling [20,21]. These areas were chosen to maximize the likelihood of recovering viable fungal elements. All samples were collected by the same examiner. One sample was obtained per animal and divided into three parts. One part was used for direct microscopy with 20% potassium hydroxide (KOH), which dissolves keratin, thereby clearing the hair and facilitating the visualization of fungal hyphae, spores, or other elements under the microscope. Another part was examined for ectoparasites using 10% KOH and lactophenol, while the third was cultured.

### 2.3. Direct Microscopical Examination of Samples

One portion of the sample was treated with 20% KOH on a glass slide mounted with cover slips for 10–30 min and examined under a light microscope at 400× total magnification to detect fungal elements such as hyphae and spores [3]. The second portion was examined for ectoparasites, including fleas, lice, ticks, and mites, using 10% KOH and lactophenol [22,23]. Ectoparasites were evaluated to assess their role as predisposing factors for fungal infections, as they contribute to skin microtrauma, increasing susceptibility to dermatophytes.

### 2.4. Isolation and Identification of Dermatophyte Species

Each sample was cultured on Sabouraud dextrose agar (SDA) with Dermasel selective supplement (Oxoid, Thermo Fisher Scientific; Basingstoke, UK), SDA containing chloramphenicol (Biomark Laboratories, Pune, Maharashtra, India), and dermatophyte test medium (DTM, HiMedia laboratories; Mumbai, India) with modified Dermato Supplement (FD176, HiMedia laboratories; Mumbai, India). Cultures were incubated at 32 °C for up to 4 weeks and examined daily for fungal growth.

Isolates were identified based on the phenotypic characteristics of the colonies on SDA (colony morphology: texture, growth rate, and surface and reverse color), color change in the DTM, and microscopic examination of colonies in lactophenol cotton blue (Loba Chemie Pvt. Ltd., Mumbai, India) mounts (size, shape, type of conidia, and modification of hyphae). The culture was considered negative if no growth occurred after four weeks of incubation. To confirm the identification and subtyping of *T. mentagrophytes*, a urea hydrolysis test was performed using Christensen’s urea agar (Oxoid, Hampshire, UK) [24]. *T. mentagrophytes* ATCC 28185 and *M. canis* ATCC 32903 reference strains were included as quality control.

The retrieved isolates were subjected to molecular identification using PCR, DNA sequencing of the ITS region of rDNA, and RFLP analysis.

### 2.5. DNA Extraction and PCR Assays

DNA was extracted from isolates using the QIAamp DNeasy Plant Mini Kit (Catalogue No. 69104, QIAGEN, Hilden, Germany) following the manufacturer’s instructions. The concentration and purity of DNA were determined using a NanoDrop 1000 spectrophotometer (Thermo Fisher Scientific, Waltham, MA, USA). PCR amplification of the ITS1-5.8S-ITS2 rDNA region of the DNA extracted from each isolate was performed using ITS1 (5′–TCC GTA GGT GAA CCT GCG G–3′) and ITS4 (5′–TCC TCC GCT TAT TGA TAT GC–3′) primers [25]. The total reaction mixture (25 μL) contained 12.5 μL of PCR master mix (Merck Genei), 1 µL (20 pmol) of each primer, 5 µL of template DNA, and 5.5 µL of nuclease-free water. Amplification was performed in a thermal cycler (Biometra T3, Gottingen, Germany) adjusted to initial denaturation at 95 °C for 5 min, followed by 35 cycles of denaturation at 95 °C for 30 s, annealing at 56 °C for 30 s, extension at 72 °C for 30 s, and final extension at 72 °C for 5 min. Aliquots (10 µL) of each PCR product were electrophoresed on 1.5% agarose gel (Applichem GmbH; Darmstadt, Germany) in Tris–borate–ethylenediaminetetraacetic acid (TBE) buffer and stained with 0.5 µg/mL ethidium bromide, visualized, and photographed using the GelDoc UV gel documentation system (Alpha Inno-Tech; San Leandro, CA, USA). A 100 bp Plus DNA molecular size marker (Qiagen) was used to determine the amplicon sizes.

#### 2.5.1. RFLP Analysis

All PCR products were digested with the endonuclease restriction enzyme *Mva*I (Fast Digest, Fermentas Life Sciences, Vilnius, Lithuania), which recognizes the sequence 5’ CC (T/A) GG 3′ at 37 °C for 20 min [26]. The reaction mixture contained 8 µL of PCR products, 0.5 µL of *Mva*I, 1.5 µL of 10X buffer, and 5 µL of molecular-grade water. Restriction fragments were detected by electrophoresis on a 1.5% agarose gel in TBE buffer, visualized, and photographed using the GelDoc UV gel documentation system. A 50 bp DNA molecular size marker (Fermentas, Vilnius, Lithuania) was used to determine the amplicon sizes.

#### 2.5.2. DNA Sequencing and Sequence Analysis

The PCR products were purified using the GeneJET PCR Purification Kit (Thermo Fisher Scientific; Fermentas, Waltham, MA, USA) and sequenced in both the forward and reverse directions using ITS1 and ITS4 primers. Sequencing was performed using an automated sequencer (ABI 3730XL DNA Analyser; Macrogen, Inc., Seoul, Republic of Korea). Meticulous quality control of sequence data was assessed using chromatogram review and bidirectional concordance by aligning forward and reverse sequences before performing a BLAST search. DNA sequence data were compared with previously published data in GenBank using the BLAST program available at the National Centre for Biotechnology Information (http://www.ncbi.nlm.nih.gov/BLAST/, accessed on 1 February 2022). Nucleotide sequences of *M. canis*, *T. mentagrophytes*, *T. verrucosum*, *T. indotineae*, *Nannizzia gypsea*, *Malassezia pachydermatis*, and *Trichosporon asahii* were deposited in GenBank (accession Nos. PP069514, PP069518, PP069746, PP069750, PP069764, PP261902, and PP261903).

### 2.6. Treatment Trials

A total of 40 animals (20 dogs and 20 cats) showing evident clinical manifestations of dermatophytosis were enrolled to investigate the efficacy of the four different treatment regimens (Supplementary Table S2) after obtaining informed consent from their owners. All animals were treated at home by their owners, returning weekly for follow-up visits.

The zoonotic significance of the disease and the necessary protective measures were fully explained to the owner. The enrolled animals were positive for mycological examination of clinical samples. A sample size calculation at a 0.05 significance level and 80% power revealed that eight animals (four dogs and four cats) per group (G) would have been required. Animals exhibiting comparable lesion severity and distribution were randomly assigned to five groups (G1–G5) after obtaining informed owner consent. Randomization was conducted using Microsoft Excel’s RAND function to generate a random sequence for allocating animals into the respective experimental groups.

Itraconazole and clotrimazole were selected to treat the infected animals because they are effective and safe. Itraconazole is the most effective systemic antifungal for dogs and cats, while clotrimazole is an efficient topical antifungal that ensures a high local concentration at the site of infection [3]. Animals in G1 were topically treated with clotrimazole (Closol spray, European Egyptian Pharmaceutical Industries, Alexandria, Egypt) twice daily for two weeks. Before application, the affected area and the surrounding skin (extending 5 to 10 cm) were carefully cleaned and moistened with the spray until visibly wet. G2 was topically treated with clotrimazole and 1% itraconazole oral suspension (Zoovet S.A., Ciudad de Santa Fe, Argentina) at a dose of 10 mg/kg body weight (1 mL/kg) on alternating weeks for three treatment cycles [27]. G3 received clotrimazole, itraconazole, and supportive treatment with multivitamins and minerals (MultiBoost, Mervue Laboratories, Watergrasshill, Co., Cork, Ireland) at a dose of 1 mL/kg body weight once daily for one month. G4 received clotrimazole, itraconazole, MultiBoost, supportive treatment, and Vacderm vaccine (Vetzverocentr, Shchelkovo Biocombinat, Shchelkovo, Russia). Vacderm vaccine (0.5 mL for cats aged 1–3 months and dogs from 2 months and weighing less than 5 kg, or 1 mL for cats ˃ 3 months and dogs from 2 months weighing more than 5 kg) was given via intramuscular injections in alternating hind limbs (thigh area) with an interval of 10–14 days. Animals in G5 were the infected, untreated control group.

Animals were clinically observed weekly over eight weeks for general health parameters and any adverse events (vomiting, salivation, anorexia, lethargy, diarrhea, and jaundice). The treatment efficacy was assessed based on clinical signs and mycological examination. A 0–3 lesion scoring system was used to quantify lesion severity [19,28] (Supplementary Table S3). The scoring of each examined area of the body and each animal’s total score was estimated [29].

Hair samples were collected weekly for fungal culture, with negative mycological status defined as two consecutive negative cultures within a two-week interval [3,30]. Clinical worsening of the infected untreated group was monitored, and animals received treatment after the observation period.

### 2.7. Data Analysis

All data were analyzed using SPSS software version 26.0 (IBM Corp., Armonk, NY, USA) and the SAS software version 8 PROC LOGISTIC procedure. Differences in the prevalence of dermatophytosis between animals and fungal species were assessed using the chi-square test (χ^2^). For evaluation of diagnostic methods, the concordance between direct microscopy and fungal culture was assessed using kappa (κ) statistics with corresponding *p*-values. Risk factors for dermatophytosis (age, sex, breed, housing, season, nutrition, deworming, hygienic measures, ectoparasite infestation, and stress factors) were analyzed using multivariate logistic regression models. Odds ratios (OR) with 95% confidence intervals (CIs) and relative risks (RRs) were reported. Interaction terms (e.g., sex × season, housing × nutrition) were initially tested but excluded from the final models due to lack of significance. Separate regression models were fitted for dogs and cats to account for species-specific differences. One-way ANOVA and Tukey’s post hoc test were used to assess changes in clinical scores over time between groups to assess the treatment outcomes. For treatment efficacy, Kaplan–Meier survival analysis was performed to estimate recovery rates across treatment groups, and survival curves were compared using the log-rank (Mantel–Cox) test. Results are expressed as mean ± standard error (SE) with 95% CI. Statistical significance was considered at *p* < 0.05.

## 3. Results

### 3.1. Clinical Presentations

**Figure 1:**
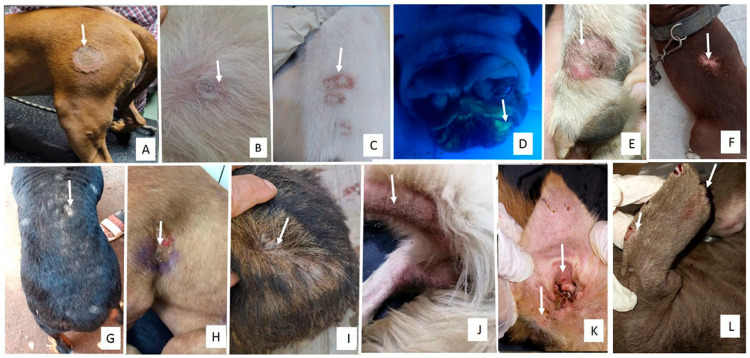
Clinical presentations of dermatomycosis in dogs. Arrow in each photo points to the lesion (**A**): Circular area of alopecia and scaling on the thigh of a 7-month-old Pitbull dog infected with *M. canis*. (**B**): Erythema, circular area of alopecia, and scaling on the abdomen of an 11-year-old Griffon infected with *M. canis*. (**C**): Multiple circular lesions on the back of a 6-month-old Griffon infected with *M. canis*. (**D**): Apple green fluorescence glow of affected hair shafts in a pug dog infected with *M. canis*. (**E**): Circular areas of alopecia with erythema, crusting, and scaling on the paw of a Husky infected with *Nannizzia gypsea*. (**F**): Erythema and alopecia on the rump of a 6-month-old Pitbull infected with *T. mentagrophytes*. (**G**): Multiple circular lesions with alopecia, crusting, and scaling of the entire back of a Rottweiler with *T. mentagrophytes*. (**H**): Crusting and hair loss behind the shoulder of a 6-month-old Mastiff infected with *T. verrucosum*. (**I**): Crusting and scaling of the lumbosacral area of an 8-month-old local breed with *T. indotineae*. (**J**): Erythema, thickening, lichenification, and hair loss of the tail of a Golden Retriever dog infected with *Malassezia pachydermatis*. (**K**): Otitis externa, hair loss, and erythema of the ear of a Golden Retriever dog infected with *M. pachydermatis*. (**L**): Ear tip fissuring with white deposits on the hair of the ear of a 7-month-old Pitbull dog infected with *Trichosporon asahii*.

**Figure 2:**
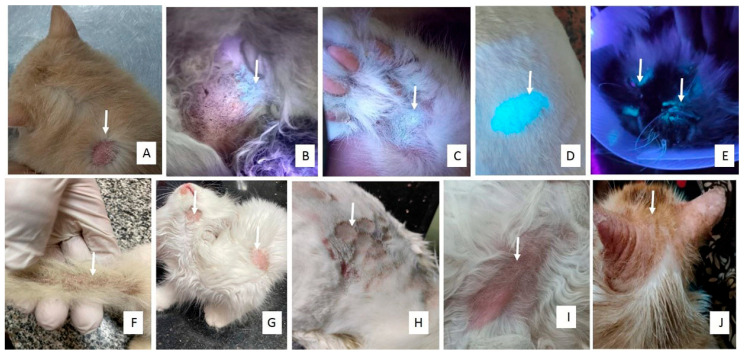
Clinical presentations of dermatophytosis in cats. Arrow in each photo points to the lesion (**A**): Circular alopecia, erythema, and scaling of the scruff of a 2-year-old female Persian cat infected with *M. canis*. (**B**): Apple green fluorescence revealed by Woods lamp examination of the abdomen of a 3-month-old Persian male cat. Wood’s lamp examination of the paw of a 2-month-old Persian kitten (**C**), abdomen of a 5-month-old Persian cat (**D**), and head of a 9-month-old Persian cat (**E**) revealed apple green fluorescence. (**F**): Stud tail of a 3-month-old Persian cat with concurrent *M. canis* infection. (**G**): Circular area of alopecia, erythema, and scaling of the chin, neck, and abdomen of a 3-month-old Persian kitten infected with *M. canis*. (**H**): Polycyclic coalescing lesions on the scruff of a 1-year-old female Persian cat with generalized dermatophytosis caused by *M. canis*. (**I**): Erythema on the abdomen of a 1-year-old male Persian cat infected with *T. mentagrophytes*. (**J**): Erythema, crusting, scaling, and alopecia of the ears and head of a 9-month-old Persian cat infected with *T. mentagrophytes*.

Dogs and cats affected by dermatophyte infections exhibited pruritus, asymmetrical, rounded lesions with peripheral erythema, and broken hairs in the early stages. In more advanced stages, the lesions expanded and coalesced into broad areas of circular alopecia with scaling, crusting, and follicular plugging (Figure 1 and Figure 2). Most lesions were located on the ears, head, and neck of kittens and the scruff of adult female cats. Cats with poor management conditions and grooming practices showed matted hair on the abdomen with dermatophyte lesions underneath the abdomen and paws. In dogs, lesions were predominantly observed on the rump, abdomen, and head.

### 3.2. Direct Microscopy and Fungal Culture of Samples

Of the 90 dog samples examined, 47 (52.22%) were positive by fungal culture (*p* = 0.655, 95% CI: 0.67–2.14), and 50 (55.56%) tested positive by direct microscopy (*p* = 0.18, 95%CI: 0.87–32.8). In cats, fungal culture and direct microscopy yielded similar results, with 35 positive samples (70%) (*p* < 0.0001, 95% CI: 2.32–12.81). The concordance between direct microscopy and culture was highly significant, moderate for dogs (*p* < 0.0001; kappa value: k = 0.53), but highly significant and perfect for cats (*p* < 0.0001; k = 1.00) (Table 1).

### 3.3. Dermatophyte Species Isolated from Dogs and Cats

*Microsporum canis* was significantly more prevalent among cats (82.86%) than dogs (44%) (*p* = 0.001, 95% CI: 0.14–0.66) (Table 2). The frequency of *T. mentagrophytes* was 26% in dogs and 11.42% in cats. Other species isolated from dogs included *T. verrucosum* and *T. indotineae*. One *T. indotineae* isolate from a dog exhibited flat and granular colonies, slightly raised in the center with a beige surface and light brown reverse. Microscopy showed spherical and pyriform microconidia, cigar-shaped macroconidia, and spiral hyphae (Supplementary Figure S1). There was a highly significant difference in the prevalence of all fungal species across dogs, cats, and total isolates (*p* < 0.0001 for each) (Table 2).

Mixed infections with *M. canis* and *T. mentagrophytes* were observed in one dog. Also, a seven-month-old Pitbull had a mixed infection with *T. asahii* (white piedra), *M. pachydermatis* on the tail and interdigital spaces, and *T. mentagrophytes* on the thighs and rump. Molecular identification via PCR-RFLP targeting the ITS region of rDNA successfully confirmed species identification. The amplified product sizes ranged from 650 to 750 bp for various species. RFLP patterns were specific for *T. mentagrophytes* (405/124/90/53 bp), *T. verrucosum* (517/141/20 bp), M. canis (441/165/103/28 bp), and N. gypsea (400/250 bp) (Supplementary Figures S2 and S3).

### 3.4. Potential Risk Factors for Dermatophytosis in Pets

Logistic regression analysis revealed that adult dogs (1–5 years and >5 years), compared with younger dogs (<1 year), exhibited a significantly lower risk of dermatophyte infection (*p* = 0.01; OR = 0.34; 95% CI = 0.22–0.73 and OR = 0.50; 95% CI = 0.29–1.04, respectively) (Table 3).

In cats, most of the variables assessed were significantly associated with ringworm (*p* < 0.05). These variables included age, sex, breed, housing environment, season, hygienic measures, and stress factors (Table 4). A significantly lower risk of infection was found in crossbreed cats (*p* = 0.01; OR = 0.30; 95% CI = 0.16–0.85) and local breed cats (*p* = 0.01; OR = 0.15; 95% CI = 0.01–0.45) when compared with Persian cats. Indoor/outdoor housing significantly reduced the risk of infection (*p* < 0.0001; OR = 0.05, 95% CI = 0.01–0.28) when compared with indoor housing.

The prevalence of dermatophyte infections in dogs non-significantly (*p* = 0.29) increased by 51.1% in winter (OR = 1.51; 95% CI = 0.82–2.35) and by 2.3 times in autumn (OR = 2.26; 95% CI = 1.76–3.79) compared to the summer season. In contrast, it decreased by 24.5% in spring (OR = 0.75; 95% CI = 0.24–1.34).

In cats, the infection rate significantly (*p* = 0.015) increased relative to the summer season (Table 4). This increase was 10-fold in winter (OR = 10; 95% CI = 5.63–14.96), 19-fold in spring (OR = 19; 95% CI = 15.32–23.96), and 2-fold in autumn (OR = 2; 95% CI = 0.86–3.26). The prevalence of dermatophyte infections was significantly higher in both dogs (*p* < 0.0001; OR = 81.37; 95% CI = 69.25–98.32) and cats (*p* < 0.001; OR = 12.45; 95% CI = 8.33–14.23) without ectoparasites compared to those with ectoparasite infestation (fleas, mites, and tick infestations) (Supplementary Figure S4). Regarding concurrent infestations, ectoparasites were identified in 17.78% of dogs and 24% of cats.

Collinearity among independent variables was assessed using the variance inflation factor (VIF), and no significant multicollinearity was detected. Additionally, interaction terms between key variables (e.g., breed × age, sex × season) were tested but were not statistically significant and therefore excluded from the final model.

### 3.5. Treatment Outcomes

The evaluation of treatment efficacy across different regimens demonstrated variable therapeutic effectiveness (Supplementary Tables S4–S7). On day 0, all 20 dogs and 20 cats presented with clinical lesions, and cultures from all animals were positive for *M. canis*. In G1, clinical improvement following topical clotrimazole treatment began during the second week. By the third week, there was a noticeable improvement in crusting lesions, although alopecia persisted (Supplementary Figure S5). By the fifth week, all cats appeared clinically normal (Supplementary Figure S6), although two cats developed new focal areas of crusting and alopecia on their heads and necks. Complete resolution of clinical lesions was achieved by the seventh week in dogs and the eighth week in cats, with negative culture results in all samples.

**Figure 3:**
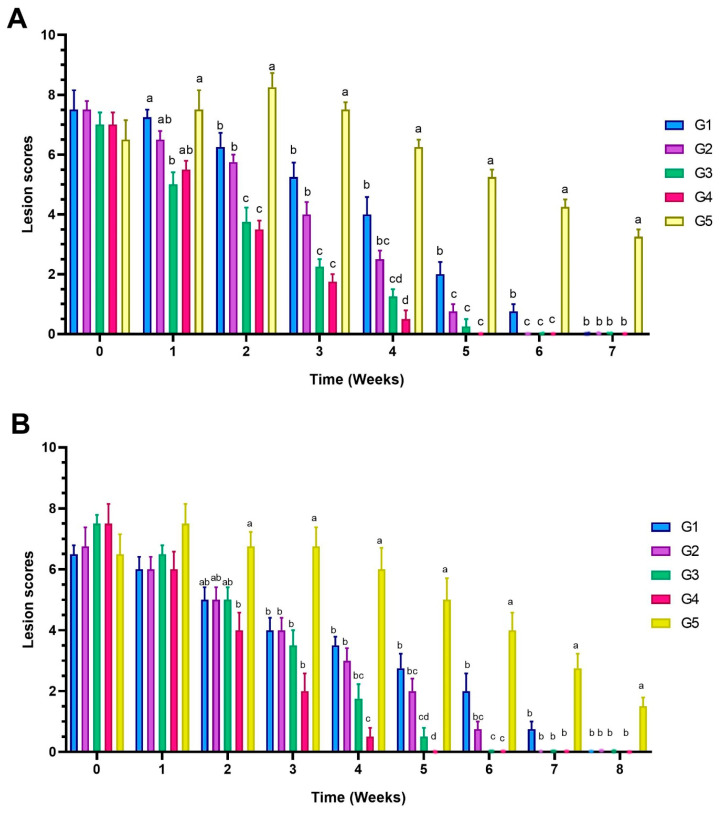
Mean score of dogs (**A**) and cats (**B**) suffering from ringworm and treated with different regimes. G1 animals were topically treated with clotrimazole, G2 were topically treated with clotrimazole and 1% itraconazole oral suspension, G3 received clotrimazole, itraconazole, and supportive treatment with multivitamins and minerals, and G4 received clotrimazole, itraconazole, supportive treatment, and vacderm vaccine. Animals in G5 were infected and untreated. Results are expressed as mean± SEM (standard error of the mean). ^a–d^ Means with various superscript letters within the same column indicate significant difference at *p* < 0.05.

The combined treatment with clotrimazole and oral itraconazole (G2) demonstrated greater efficacy, with clinical and mycological cure achieved by the sixth week in dogs and the seventh week in cats (Figure 3). Improvement was noticeable from the second week of treatment (Supplementary Figures S7 and S8), and culture results were consistently negative by the time of clinical resolution.

**Figure 4:**
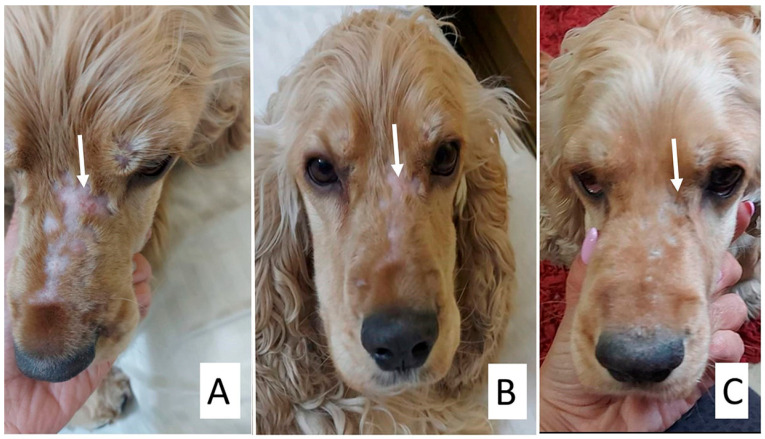
Treatment of a Cocker Spaniel dog with clotrimazole, itraconazole, and supportive treatment. The arrow in each photo points to the lesion. (**A**) Areas of alopecia on face, (**B**) 2 weeks post-treatment, and (**C**) 4 weeks post-treatment.

The addition of supportive treatment (G3) further enhanced therapeutic outcomes, with hair regrowth and marked improvement in alopecia observed within ten days (Figure 4). Complete clinical and mycological resolution occurred by the fifth week in dogs and the sixth week in cats, with all culture results turning negative.

**Figure 5:**
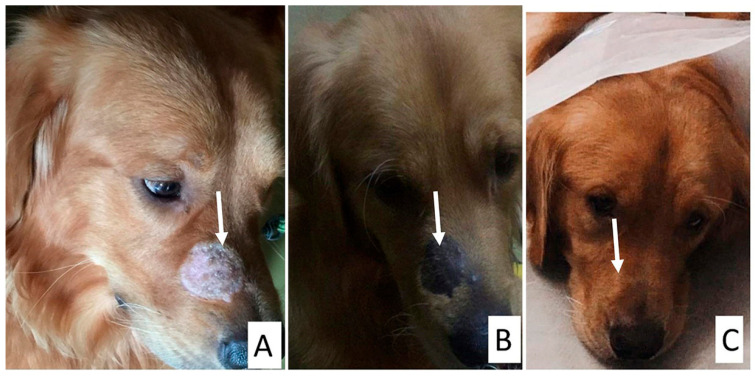
Treatment of a four-year-old Golden Retriever dog with clotrimazole, itraconazole, supportive treatment, and vacderm vaccine. The arrow in each photo points to the lesion. (**A**) Circular area of alopecia and scales on muzzle, (**B**) 2 weeks post-treatment, and (**C**) 4 weeks post-treatment.

**Figure 6:**
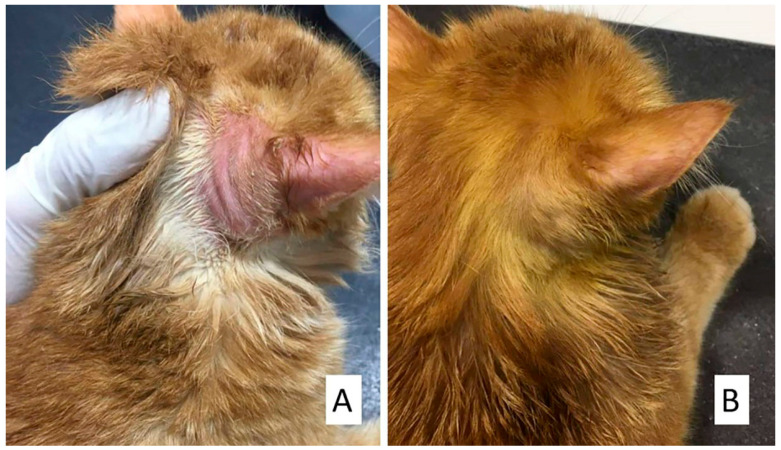
Treatment of a 5-year-old Persian female cat with clotrimazole, itraconazole, supportive treatment, and vacderm vaccine. (**A**) Alopecia and erythema on the ear and neck, (**B**) 4 weeks post-treatment.

Treatment with clotrimazole, itraconazole, supportive therapy, and the vacderm vaccine (G4) demonstrated the most rapid clinical improvement, with the noticeable resolution of lesions within the first week (Figure 5 and Figure 6). Complete clinical and mycological resolution was achieved by the fourth week in dogs and the fifth week in cats (Figure 3). Culture results were consistently negative in all treated animals. No clinically evident hepatotoxicity was observed during the treatment period.

The untreated control group (G5) exhibited a normal disease progression, with initial worsening of clinical signs followed by gradual recovery over time. Mild improvement occurred over time, but without a negative mycological status (Supplementary Figure S9 and Supplementary Tables S4–S7).

Statistical analysis revealed significant (*p* < 0.01) variation between treated dogs and control untreated ones at 1, 2, 3, 4, 5, 6, and 7 weeks post-treatment. At 4 weeks post-treatment, dogs in group G4 showed the most significant (*p* < 0.0001) decrease in the score of ringworm in infected dogs when compared with the control infected untreated dogs. At 5 weeks post-infection, dogs in treatment group G4 showed the most significant (*p* < 0.0001) decrease in the score of ringworm lesions in infected dogs (0), followed by G3 (0.25) and G2 (0.75) groups, when compared with the control infected untreated dogs, with no significant difference between G2, G3, and G4 groups (Figure 3 and Supplementary Table S6).

Statistical analysis revealed significant (*p* < 0.05) variation between treated cats and control untreated ones at 2, 3, 4, 5, 6, 7, and 8 weeks post-treatment. Cats in treatment groups G3 and G4 showed the most significant (*p* < 0.0001) decrease in the score of ringworm lesions in infected cats when compared with the control infected untreated cats, with no significant difference between G3 and G4 groups (Figure 3 and Supplementary Table S7).

**Figure 7:**
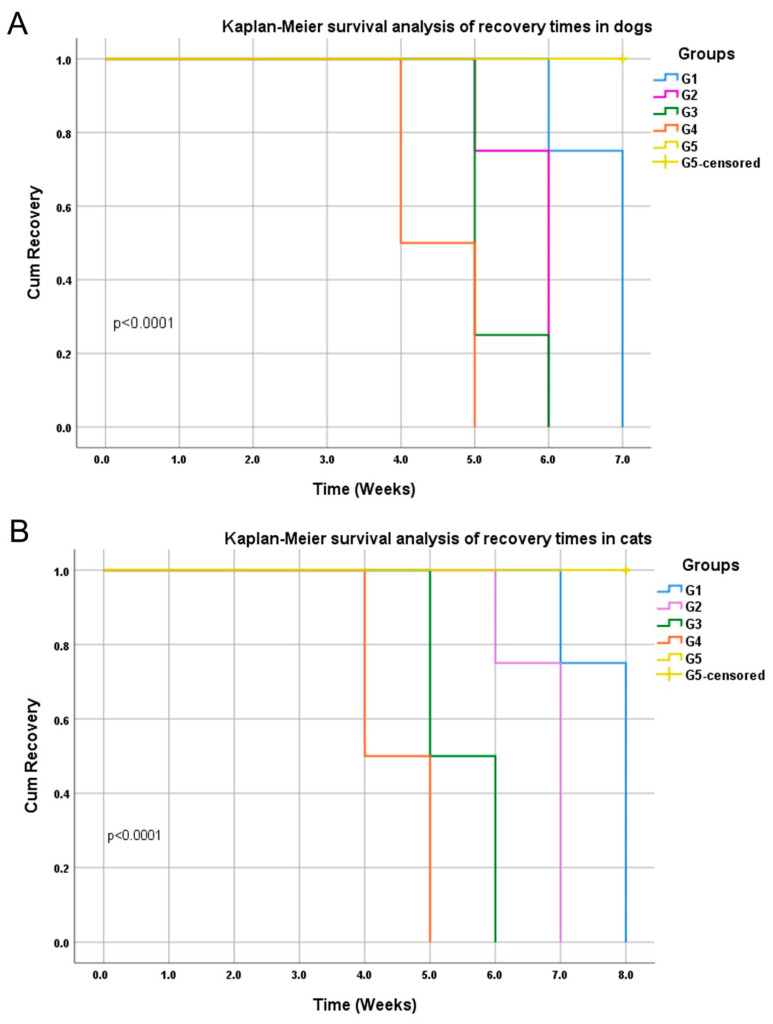
Kaplan–Meier survival analysis of recovery times in the different treatment regimens for infected dogs (**A**) and cats (**B**). G1 animals were topically treated with clotrimazole, G2 was topically treated with clotrimazole and 1% itraconazole oral suspension, G3 received clotrimazole, itraconazole, and supportive treatment with multivitamins and minerals, and G4 received clotrimazole, itraconazole, supportive treatment, and vacderm vaccine. Animals in G5 were infected and untreated. Highly significant differences were detected between the recovery time in the treated groups (*p* < 0.0001). Censored referred to animals in the control group (G5) that did not reach full recovery at the end of the observation period.

Kaplan–Meier survival analysis of recovery time and log-rank test revealed highly significant differences in the treated groups with the rapid recovery of ringworm lesions in G4 (*p* < 0.0001) (Figure 7).

## 4. Discussion

Accurate diagnosis of dermatophytosis and yeast infections in pet animals and the rapid allocation of appropriate treatment are essential to shorten recovery time and limit the spread of infection to other animals and humans [3,31]. This study investigated the prevalence, diagnostic methods, and risk factors of dermatophyte infections in dogs and cats, as well as identified an effective, short-duration treatment protocol.

The concordance analysis between direct microscopy and fungal culture revealed moderate agreement in dogs but perfect agreement in cats. This discrepancy may be related to differences in sample quality, lesion characteristics, or species-specific factors. While fungal culture remains the gold standard for accurate diagnosis, microscopy as a rapid screening tool provides valuable preliminary information to guide initial treatment decisions [16,20,32].

Molecular methods, such as PCR-RFLP, are rapid and reliable confirmatory tests for the results of culture-based tests based on phenotypic features for identifying dermatophyte species [33]. This study employed PCR-RFLP targeting the ITS region of rDNA for species identification, demonstrating efficacy in accurately differentiating dermatophyte species. Molecular methods are indispensable for confirming species identity, especially for emerging pathogens like *T. indotineae*, which may not be easily distinguishable through conventional techniques [34,35]. Accurate identification is crucial for effective treatment planning and epidemiological tracking [36]. The amplified product sizes of the ITS regions were species-specific [16,37,38]. PCR-RFLP offers a reliable and rapid confirmatory method for identifying dermatophyte species, which is particularly relevant for emerging species like *T. indotineae* [38,39].

The overall prevalence of dermatophytes and yeasts isolated from dogs and cats was 58.57% (82/140), with 52.22% (47/90; *p* = 0.65) in dogs and 70% (35/50; *p* < 0.0001) in cats. This prevalence rate is higher than reported in studies from Nigeria (49.5% in dogs and 61% in cats) [40], Italy (20.5% in dogs and 28.2% in cats) [41], Bulgaria (12.9% in dogs and 13.5% in cats) [42], and Russia (4.5% in dogs and 9.5% in cats) [43]. Also, previous studies in Egypt reported prevalence rates of 51.4% in cats [44], 20.4% in dogs, and 16.1% in cats [45]. However, it is lower than the findings from Azerbaijan [46]. The geographical distribution, climate, temperature, humidity, housing environment, population sampled, and the presence of natural reservoirs likely influence prevalence rates [47].

The predominance of *M. canis* as the primary dermatophyte isolated, especially in cats (82.86%), aligns with prior studies emphasizing its zoonotic potential and high transmissibility in both household and shelter environments [48]. For instance, research indicates that *M. canis* is responsible for 81.8% to 97% of dermatophytosis cases in pets [2,49]. This underscores the necessity for effective diagnostic and treatment strategies to reduce the risk of zoonotic transmission [50,51].

The predominance of *M. canis* (60%), followed by *T. mentagrophytes* (20%), *Nannizzia gypsea* (2.35%), and *T. verrucosum* (5.88%), aligns with previous studies [52]. Transmission from infected cats to owners was confirmed in five cases, emphasizing the public health implications of *M. canis* infections [53].

In this study, we isolated the dysgonic *M. canis* var. *distortum*, characterized by its distinctively distorted and roughened macroconidia, from a stray cat. Previously found in cats [54], this strain was also responsible for a dermatophytosis outbreak transmitting from stray cats to dogs and humans [55]. Additionally, we identified *T. indotineae* from a dog for the first time in Africa, highlighting its potential to spread beyond human populations. The primary hosts for *T. indotineae* are humans, as confirmed by hundreds of infections in patients worldwide [56]. Although cases of infected animals with *T. indotineae* are still limited [10,11], extending the host range of *T. indotineae* from humans to animals is a big issue that the world could face in the next generation [57]. Emerging terbinafine resistance in dermatophytes, particularly in *T. indotineae*, has become a significant concern worldwide. This resistance is primarily associated with mutations in the squalene epoxidase (SQLE) gene [58]. Recent multicountry studies [49,57,59,60] underscore the critical importance of One Health surveillance for pet dermatophytosis, demonstrating the interconnectedness of animal, human, and environmental health. Surveillance is key to tracking the prevalence of dermatophyte species and identifying high-risk areas. By understanding dermatophytosis prevalence in different regions, health authorities can develop targeted educational campaigns for pet owners and veterinarians. This knowledge also provides more effective treatment and prevention guidelines, thereby protecting both animal and human populations. The findings on species prevalence and risk factors are crucial for informing national and international public health strategies to control the spread of dermatophytes [60].

In the present study, age was significantly associated with dermatophyte infections in both dogs and cats. Dogs younger than one year, compared with adult animals (>1 year), had a significant (*p* = 0.01) prevalence of dermatophytosis. This could be related to immunological immaturity and deficiency of fungistatic sebum or linoleic acid [3,31]. Moreover, ringworm in cats older than one year may be attributed to several factors, including immunosuppression (due to inappropriate or prolonged corticosteroid therapy or long-term antibiotic treatment), other concomitant systemic diseases, pregnancy, and nutritional deficits (especially in proteins and vitamin A) [3,49].

There was no apparent sex predisposition in the dogs. This finding is similar to that previously reported by Cabañes et al. [52]. However, other studies have reported a higher prevalence of dermatophytes in male dogs than in females, suggesting possible population- or environment-related variation [41]. In the present study, small breeds of dogs had a higher prevalence of dermatophytosis than the large breeds. In addition, Persian cats had the highest prevalence compared to other breeds. The small sample size limits the generalizations. Similar findings have been reported by Moretti et al. [12], which are likely due to the long-haired nature of these breeds, which may facilitate spore adherence and reduce grooming efficacy. Regarding the housing environment, dogs outdoors had a slightly higher prevalence of dermatophytosis than those indoors. This may be due to more contamination in the environment and the presence of rodents, which can help spread infections [3,61].

Regarding cats, indoor/outdoor housing significantly reduced the risk of infection (*p* < 0.0001; OR = 0.05, 95% CI = 0.01–0.28) when compared with indoor housing. This agrees with the results of a previous study [62]. Overcrowded indoor environments may contribute to fungal spore transmission, especially under poor hygiene and elevated humidity. In addition, using contaminated clippers or tools in grooming pet shops leads to microtrauma, which predisposes to dermatophytosis. Nevertheless, other studies have reported that outdoor transmission is a significant infection route for ringworm [63,64].

The analysis of treatment outcomes provided valuable insights into the efficacy of different therapeutic regimens. The combination of topical clotrimazole, oral itraconazole, supportive therapy, and the vacderm vaccine demonstrated the highest efficacy, achieving clinical and mycological resolution within four weeks in dogs and five weeks in cats (*p* < 0.0001). The rapid response observed with this regimen may be attributed to the synergistic effects of topical and systemic antifungal agents, immune support, and vaccination-induced enhancement of host defenses. Supportive therapy, including immunostimulants, appeared to contribute to faster recovery and higher cure rates, highlighting the importance of addressing host immunity in managing dermatophytosis [3]. Similarly, Shadskaya confirmed the effect of this protocol, which also included etiotropic drugs of systemic and local action (dermicocide and fungin, mycozoral 2%), immunostimulants (imunofan, NPP BIONOKS, Moscow, Russia), and complex vitamin preparations (Trivet Pharmaceuticals Pvt. Ltd., Karnal, India), which was a comprehensive treatment regimen effective for the treatment of cats and dogs and for the rapid recovery of cases [65]. Antifungal vaccines might not prevent initial infection, but they could be helpful as an additional treatment [3]. Mihaylov et al. demonstrated the effects of using the vacderm vaccine (Shchelkovo Biocombinat, Shchelkovo, Russia) in seven dogs and three cats with signs of generalized dermatophytosis [42]. They found that by the end of the third week post-treatment, there were no skin lesions, and the culture results were negative.

Comparatively, monotherapy regimens were less effective, with longer resolution times and higher recurrence rates [66,67]. These findings support the use of combination therapy to enhance treatment efficacy and reduce disease duration. Additionally, the untreated control group exhibited only mild improvement over time, emphasizing the necessity of prompt and appropriate intervention. Kurtdede et al. reported that topical clotrimazole and systemic itraconazole were effective against dermatophytosis, and clinical improvement occurred within a month [68].

The limitation that should be acknowledged in this study is the relatively small sample size for treatment evaluation. This may limit the generalizability of the findings, particularly concerning the efficacy of combination therapy.

## 5. Conclusions

This study provides valuable insights into dermatophytosis in affected dogs and cats, focusing on its prevalence, risk factors, and treatment outcomes. Our findings highlight the importance of accurate diagnosis, effective treatment, and continued surveillance to help reduce the risks of zoonotic transmission and the emergence of drug-resistant fungal species. Notably, *M. canis* was the predominant species identified in affected animals. The detection of *T. indotineae* in a dog in Egypt highlights the urgent need for heightened awareness and further research into its epidemiology and resistance patterns. Given the zoonotic nature of *M. canis* and the emerging threat of *T. indotineae*, these findings are highly relevant for One Health and veterinary public health.

Treatment outcomes revealed that combined systemic itraconazole, topical clotrimazole, supportive therapy, and a dermatophyte vaccine resulted in the fastest clinical recovery. It is important to acknowledge that the small sample size and the lack of blood sampling to monitor hepatic enzymes before and after treatment limit the generalizability of these findings.

## Figures and Tables

**Table 1:** Comparison of the results of direct microscopical examination and fungal culture of dog and cat samples.

Test	Fungal Culture		Concordance (k), (95% CI)	*p*-Value
	Negative	Positive		
Direct microscopical examination				
**In dogs**				
Negative	30/42 (71.43)	9/47 (19.15)	0.53 (0.35–0.70)	<0.0001
Positive	12/42 (28.57)	38/47 (80.85)		
**In cats**				
Negative	15/15 (100)	0/35 (0)	1 (1–1)	<0.0001
Positive	0/15 (0)	35/35 (100)		

The numbers in parentheses represent percentages of the total for each category.

**Table 2:** Frequency of dermatophyte species and yeasts isolated from dogs and cats.

Fungal Species	No. of Fungal Isolates (%)		*p*-Value ^a^, (95% CI)	Total no. of Fungal Isolates (*n* = 85)
	Dogs (*n* = 50)	Cats (*n* = 35)		
*Microsporum canis*	22 (44)	29 (82.86)	0.001 **, (0.14–0.66)	51 (60)
*Microsporum canis var distortum*	-	1 (2.86)	0.412, (0.92–1)	1 (1.18)
*Nannizzia gypsea (Microsporum gypseum)*	1 (2)	1 (2.86)	1, (0.93–1.1)	2 (2.35)
*Trichophyton mentagrophytes*	13 (26)	4 (11.42)	0.167, (0.98–1.5)	17 (20)
*Trichophyton verrucosum*	5 (10)	-	0.074, (1–1.2)	5 (5.88)
*Trichophyton indotineae*	1 (2)	-	1, (0.98–1.1)	1 (1.18)
*Malassezia pachydermatis*	6 (12)	-	0.077, (1.1–1.3)	6 (7.06)
*Trichosporon asahii*	2 (4)	-	0.51, (0.98–1.1)	2 (2.35)
*p*-value ^b^, (95% CI)	<0.0001, (0–0.01)	<0.0001, (0–0.01)		<0.0001, (0–0.04)

^a^ Indicate significant difference in the prevalence of each fungal species between dogs and cats; ^b^ Indicate significant difference in the prevalence of all investigated fungal species across dogs, cats, and total isolates. ** indicates high significant difference.

**Table 3:** Logistic regression model of potential risk factors associated with the prevalence of dermatophyte infections in dogs.

Risk Factor	Yes	No	Prevalence (%)	Β ^a^	S.E. (β)	OR ^b^ (95% CI)	RR (95% CI)
Age (*p* = 0.01)							
<1 (ref.)	24	12	66.67				
1–5	17	25	40.47	−0.78	0.46	0.34 (0.22–0.73)	0.61 (0.38–0.97)
>5	6	6	50	−0.57	0.32	0.50 (0.29–1.04)	0.75 (0.36–1.16)
Sex (*p* = 0.57)							
Male (ref.)	29	29	50				
Female	18	14	56.25	0.25	0.14	1.28 (0.54–2.06)	1.13 (0.84–1.52)
Breed (*p* = 0.19)							
Large (ref.)	34	36	48.57				
Small	13	7	65	0.54	0.17	0.19 (1.53–2.63)	1.34 (1.04–1.89)
Housing environment (*p* = 0.97)							
Indoor (ref.)	21	19	52.50				
Outdoor	17	15	53.13	0.03	0.01	1.03 (0.70–1.60)	1.01 (0.88–1.36)
Indoor/outdoor	9	9	50	−0.10	0.06	0.90 (0.59–1.75)	0.95 (0.66–1.20)
Season (*p* = 0.29)							
Summer (ref.)	15	17	46.88				
Winter	8	6	57.14	0.41	0.14	1.51 (0.82–2.35)	1.22 (0.89–1.43)
Spring	8	12	40	−0.28	0.17	0.75 (0.24–1.34)	0.85 (0.60–1.02)
Autumn	16	8	66.67	0.82	0.56	2.26 (1.76–3.79)	1.42 (1.23–1.72)
Nutrition (*p* = 0.95)							
Good (ref.)	20	18	52.63				
Poor	27	25	51.92	−0.03	0.01	0.97 (0.42–1.55)	0.98 (0.77–1.12)
Deworming (*p* = 0.41)							
Yes (ref.)	27	21	56.25				
No	20	22	47.62	−0.35	0.124	0.71 (0.41–1.62)	0.85 (0.63–1.19)
Hygienic measures and grooming practices (*p* = 0.02)							
Good (ref.)	15	5	75				
Poor	32	38	45.71	−1.27	0.57	0.28 (0.09–0.85)	0.61 (0.42–0.90)
Ectoparasites (*p* < 0.0001)							
Yes (ref.)	16	42	27.59				
No	31	1	96.88	4.39	1.05	81.37 (69.25–98.32)	3.51 (1.32–1.59)
Stress factors (*p* = 0.97)							
Infectious	9	8	52.94				
Noninfectious	24	22	52.17	−0.024	0.07	0.97 (0.75–1.18)	0.98 (0.63–1.32)
Negative	14	13	51.85	−0.011	0.002	0.96 (0.64–1.39)	0.98 (0.58–1.40)

^a^ Regression coefficient; ^b^ odds ratio; RR, relative risk; CI, confidence interval; Ref., reference comparison group in the logistic regression analysis.

**Table 4:** Logistic regression model of potential risk factors associated with the prevalence of dermatophytes infection in cats.

Risk Factor	Yes	No	Prevalence (%)	β ^a^	S.E. (β)	OR ^b^ (95% CI)	RR (95% CI)
Age (*p* = 0.02)							
<1(ref.)	19	11	63.33				
>1	16	4	80	0.86	0.39	2.35 (1.47–4.14)	1.26 (0.88–1.71)
Sex (*p* = 0.05)							
Male (ref.)	10	2	83.33				
Female	25	13	65.79	−0.955	0.55	0.38 (0.07–0.72)	0.78 (0.42–1.54)
Breed (*p* = 0.01)							
Persian (ref.)	30	9	76.92				
Crossbreed	4	4	50	−1.20	0.80	0.30 (0.16–0.85)	0.65 (0.22–1.17)
Local breed	1	2	33.33	−1.89	1.28	0.15 (0.01–0.45)	0.43 (0.15–0.79)
Housing environment (*p* < 0.0001)							
Indoor (ref.)	26	2	92.86				
Indoor/outdoor	9	13	40.91	−2.93	0.85	0.05 (0.01–0.28)	0.44 (0.23–0.79)
Season (*p* = 0.01)							
Summer (ref.)	1	2	33.33				
Winter	5	1	83.33	2.30	1.64	10 (5.63–14.96)	2.50 (1.43–3.37)
Spring	19	2	90.48	2.94	1.43	19 (15.32–23.96)	2.71 (1.64–4.76)
Autumn	10	10	50	0.69	0.30	2 (0.86–3.26)	1.50 (0.98–2.23)
Nutrition (*p* = 0.31)							
Good (ref.)	23	12	65.71				
Poor	12	3	80	0.74	0.24	2.08 (0.49–4.85)	1.22 (0.71–1.81)
Deworming (*p*= 0.41)							
Yes (ref.)	11	3	78.57				
No	24	12	66.67	−0.61	0.32	0.55 (0.13–2.23)	0.85 (0.67–1.41)
Hygienic measures and grooming practices (*p* = 0.26)							
Good (ref.)	20	6	76.92				
Poor	15	9	62.50	−0.69	0.33	0.50 (0.15–1.71)	0.81 (0.55–1.34)
Ectoparasites (*p* = 0.001)							
Yes (ref.)	12	13	48				
No	23	2	92	2.52	0.84	12.45 (8.33–14.23)	1.92 (1.12–2.69)
Stress factors (*p* = 0.001)							
Infectious	2	1	66.66				
Noninfectious	26	2	92.85	2.84	1.21	6.50 (4.43–8.38)	1.39 (1.093–1.839)
Negative	7	12	36.84	−1.14	0.73	0.29 (0.08–0.43)	0.55 (0.38–0.71)

^a^ Regression coefficient; ^b^ odds ratio; RR, relative risk; CI, confidence interval; Ref., reference comparison group in the logistic regression analysis.

## Data Availability

All data generated or analyzed during this study are included in this article and its supplementary information files. The relevant accession numbers for sequence data that have been deposited in the GenBank were PP069514, PP069518, PP069746, PP069750, PP069764, PP261902, and PP261903 for *M. canis*, *T. mentagrophytes*, *T. verrucosum*, *T. indotineae*, *N. gypsea*, *M. pachydermatis*, and *T. asahii*, respectively.

## Supplementary Materials

The following supporting information can be downloaded at https://www.mdpi.com/article/10.3390/ani15172622/s1: Figure S1: *T. indotineae* causing ringworm in an eight-month-old local breed dog; Figure S2: Agarose gel electrophoresis for the amplified products of ITS region of fungal isolates; Figure S3: *Mva*1 RFLP patterns of dermatophyte species and yeasts ITS amplicons; Figure S4: Ectoparasites detected in the examined animals; Figure S5: Treatment of a Pitbull dog with clotrimazole twice daily for 2 weeks; Figure S6: Treatment of a 1-year-old Persian female cat with clotrimazole; Figure S7: Treatment of a Golden Retriever dog with clotrimazole and itraconazole; Figure S8: Treatment of a 7-year-old Persian male cat with clotrimazole and itraconazole; Figure S9: Infected Yorkshire Terrier dog (a member of the control untreated group); Table S1: Demographic data of the examined animals; Table S2: Treatment protocols for 40 animals (20 dogs and 20 cats) had dermatophytosis; Table S3: Scoring dermatophytosis lesions in dogs and cats [19,28]; Table S4. Clinical scoring and outcome of treatment of dogs naturally infected with *Microsporum canis* [29]; Table S5: Clinical scoring and outcome of treatment of cats naturally infected with *Microsporum canis* [29]; Table S6: Mean score of *Microsporum canis*-infected and treated dogs in different treatment groups; and Table S7: Mean score of *Microsporum canis*-infected and treated cats in different treatment groups.

## Abbreviations

The following abbreviations are used in this manuscript:

CI	Confidence interval
DTM	Dermatophyte test media
ITS	Internal transcribed spacer region
KOH	Potassium hydroxide
OR	Odds ratio
PCR	Polymerase chain reaction
RFLP	Restriction fragment length polymorphism
RR	Relative risk
SDA	Sabouraud dextrose agar
S.E.	Standard error
VIF	variance inflation factor
