# Supplementary material for: Molecular Diagnostics and Control of Zoonotic Dermatophytosis: First Detection of Trichophyton indotineae in a Dog in Africa
Source: Animals (Basel). 2025 Sep 7;15(17):2622. doi: 10.3390/ani15172622 (PMC12427446; doi:10.3390/ani15172622)
Supplement: Supplementary file 1 [file animals-15-02622-s001.zip › Supplementary Materials.pdf]

Supplementary Material

Molecular Diagnostics and Control of Zoonotic Dermatophytosis: First Detection of *Trichophyton indotineae* in a Dog in Africa

Hend A. Zineldar[1], Wafaa M. El-Neshwy [1], Romeo T. Cristina[2], Nasser Z. Abouzeid [1], Mohammed I. Eisa [1], Florin Muselin[2*], Eugenia Dumitrescu[2], Adel Abdelkhalek[3] and Yasmine H. Tartor [4*]

[1]Animal Infectious Diseases Department, Faculty of Veterinary Medicine, Zagazig University, Zagazig 44511, Egypt.

[2]Pharmacology and Toxicology Department, Faculty of Veterinary Medicine, University of Life Sciences "King Mihai I" from Timisoara, Calea Aradului 119, 300645 Timisoara, Romania

[3]Food safety, Hygiene and Technology Department, Faculty of Veterinary Medicine, Badr University in Cairo (BUC), Badr City 11829, Egypt

[4]Microbiology Department, Faculty of Veterinary Medicine, Zagazig University, Zagazig 44511, Egypt

***Corresponding authors** E-mail : yasminehtartor@zu.edu.eg; florinmuselin@usvt.ro

Supplementary Table S1. Demographic data of the examined animals.

		No. (%) of animals		
**Parameter**	**Categories**	**Dogs (*n*=90)**	**Cats (*n*=50)**	**Total (*n*=140)**
**Locality**	Sharkia	45 (50.00)	24 (48.00)	69 (49.29)
	Giza	11 (12.22)	4 (8.00)	15 (10.71)
	Kafrelsheikh	4 (4.44)	4 (8.00)	8 (5.71)
	Cairo	18 (20.00)	10 (20.00)	28 (20.00)
	Alexandria	6 (6.67)	6 (12.00)	12 (8.57)
	Ismailia	6 (6.67)	2 (4.00)	8 (5.71)
**Age**	<1	36 (40)	30 (60.00)	66 (47.14)
	1 -5	42 (46.67)	18 (36.00)	60 (42.86)
	>5	12 (13.33)	2 (4.00)	14 (10.00)
**Sex**	Male	58 (64.44)	12 (24.00)	70 (50.00)
	Female	32 (35.56)	38 (76.00)	70 (50.00)
**Breed**				
	German Shepherd	18 (20.00)		
**Large Dog Breeds**	Local breed	15 (16.67)		
	Golden Retriever	12 (13.33)		
	Pit Bull	10 (11.11)		
	Husky	4 (4.44)		
	Chow Chow	3 (3.33)		
	Labrador Retriever	3 (3.33)		
	Rottweiler	2 (2.22)		
	Dogo Argentino	1 (1.11)		
	Mastiff	1 (1.11)		
	Cane Corso	1 (1.11)		
**Small Dog Breeds**	Griffons	10 (11.11)		
	Pug	1 (1.11)		
	Yorkshire Terriers	4 (4.44)		
	Cocker Spaniels	3 (3.33)		
	Pikinwah	1 (1.11)		
	Japanese Spitz	1 (1.11)		
**Cat breeds**	Domestic short-haired		3 (6.00)	
	Persian		39 (78.00)	
	Crossbreed		8 (16.00)	
**Season**				
**Winter**	December	5 (5.56)	2 (4.00)	7 (5.00)
	January	7 (7.78)	3 (6.00)	10 (14.29)
	February	2 (2.22)	1 (2.00)	3 (2.14)
	**Total**	14 (15.56)	6 (12.00)	20 (14.29)
**Spring**	March	4 (4.44)	12 (24.00)	16 (11.43)

	April	1 (1.11)	1 (2.00)	2 (1.43)
	May	15 (16.67)	8 (16.00)	23 (16.43)
	Total	20 (22.22)	21 (42.00)	41 (45.56)
**Summer**	June	9 (10.00)	-	9 (6.43)
	July	9 (10.00)	-	9 (6.43)
	August	14 (15.56)	3 (6.00)	17 (12.14)
	**Total**	32 (35.36)	3 (6.00)	35 (25.00)
**Autumn**	September	5 (5.56)	5 (10.00)	10 (7.14)
	October	16 (17.78)	6 (12.00)	22 (15.71)
	November	3 (3.33)	9 (18.00)	12 (8.57)
	**Total**	24 (26.67)	20 (40.00)	44 (31.43)
**Housing**	Mainly Indoors	40 (44.44)	28 (56.00)	68 (48.58)
	Indoors/Outdoors	18 (20.00)	22 (44.00)	40 (28.58)
	Mainly Outdoors	32 (35.56)	-	32 (22.86)
**Nutrition**	Good	38 (42.22)	35 (70.00)	73 (52.14)
	Poor	52 (57.78)	15 (30.00)	67 (47.86)
**Deworming**	Yes	48 (53.33)	14 (28.00)	62 (44.29)
	No	42 (46.67)	36 (72.00)	78 (55.71)
**Hygienic measures**	Good	20 (22.22)	26 (52.00)	46 (32.86)
	Poor	70 (77.78)	24 (48.00)	94 (65.71)
**Concurrent stress**	Infectious causes	17 (18.89)	3 (6.00)	20 (14.29)
	Non-infectious causes	46 (51.11)	28 (56.00)	74 (52.86)
	Negative	27 (30.00)	19 (38.00)	46 (32.86)

The values presented within parentheses represent percentages of the total for each category.

Supplementary Table S2. Treatment protocols for 40 animals (20 dogs and 20 cats) had dermatophytosis.

*Group	Drugs	Dose	Route	Frequency	Duration
**1**	Clotrimazole	Twice daily	Topical	Twice daily	2 weeks
**2**	Clotrimazole	Twice daily	Topical	Twice daily	2 weeks
	Itraconazole	5-10 mg/kg (1 mL/ kg)	Oral	Once daily for 7 days, then on alternating week for three treatment cycles	
**3**	Clotrimazole	Twice daily	Topical	Twice daily	2 weeks
	Itraconazole	10 mg/kg (1 mL/ kg)	Oral	Once daily for 7 days, then on alternating week for three treatment cycles	
	MultiBoost	1 ml/kg	Oral	Once daily	One month
**4**	Clotrimazole		Topical	Twice daily	2 weeks
	MultiBoost	1 ml/kg	Oral	Once daily	One month
	Itraconazole	1 mL/ kg	Oral	Once daily for 7 days, then on alternating week for three treatment cycles.	
	Vacderm vaccine	0.5 mL for cats 1-3 months and dogs < 2 months or < 5 kg.<br>1 mL for cats > 3 months and dogs > 2 months or > 5 kg	IM	Two doses with 10-14 days interval.	
**5**	Control	Non treated			

*Each group contains four dogs and four cats. IM: intramuscular.

Supplementary Table S3. Scoring dermatophytosis lesions in dogs and cats according to Moriello *et al.* (2004) and Puls *et al.* (2018)

Score index	Score 0	Score 1	Score 2	Score 3
**Alopecia**	Non	Mild with marked hair growth	Moderate with partial hair loss	Severe or complete hair loss
**Erythema**	Non	Faint red; noticeable only on close examination	Bright red; easily visible upon close examination	Severe erythema; easily visible from a 1-meter distance
**Scaling and crusting**	Non	Fine, mild scaling, noticeable only on close examination	Easily visible scaling, minimal crust	Severe, large, and extensive crusting with keratinization
**Induration**	Nonvisible	Noticeable only upon close examination and palpation of lesion	Easily palpable but not visible	Easily palpable and visible

Moriello, K.A.; Deboer, D.J.; Schenker, R.; Blum, J.L.; Volk, L.M. Efficacy of pre-treatment with lufenuron for the Prevention of *Microsporum canis* infection in a feline direct topical challenge model. *Vet Dermatol* **2004**, *15*, 357–362, doi:10.1111/J.1365-3164.2004.00406.X.

Puls, C.; Johnson, A.; Young, K.; Hare, J.; Rosenkrans, K.; Young, L.; Moriello, K. Efficacy of itraconazole oral solution using an alternating-week pulse therapy regimen for treatment of cats with experimental *Microsporum canis* infection. *J Feline Med Surg* **2018**, *20*, 869–874, doi:10.1177/1098612X17735967.

Supplementary Table S4. Clinical scoring and outcome of treatment regimens for treatment of dogs naturally infected with *Microsporum canis*

Group	Dog	Clinical scoring								Clinical outcome	
		Week 0	Week 1	Week 2	Week 3	Week 4	Week 5	Week 6	Week 7	Recovery	Relapse
**G 1**	D1	(6)<br>I 1, E1, A 2, S2<br>One lesion on the back.	(5)<br>I 1, E0, A 2, S2	(5)<br>I1, E0, A 2, S2	(4)<br>I1, E0, A 2, S1	(3)<br>I1, E0, A2, S0	(1)<br>I1, E0, A0, S0	(0)		Week 6	ND
	D2	(9)<br>I1, E2, A 3, S3<br>One lesion on the trunk.	(8)<br>I1, E1, A 3, S3	(7)<br>I1, E0, A 3, S3	(6)<br>I1, E0, A 3, S2	(5)<br>I1, E0, A 2, S2	(3)<br>I1, E0, A 2, S0	(1)<br>I1, E0, A0, S0	(0)	Week 7	ND
	D3	(7)<br>I1, E1, A3, S2<br>One lesion on the tail.	(6)<br>I1, E0, A3, S2	(6)<br>I1, E0, A3, S2	(5)<br>I1, E0, A2, S2	(4)<br>I1, E0, A2, S1	(2)<br>I1, E0, A1, S0	(1)<br>I1, E0, A0, S0	(0)	Week 7	ND
	D4	(8)<br>I3, E0, A3, S2<br>One lesion on the rump and shoulder.	(7)<br>I3, E0, A3, S1	(6)<br>I3, E0, A2, S1	(5)<br>I3, E0, A2, S0	(3)<br>I2, E0, A1, S0	(2)<br>I2, E0, A0, S0	(1)<br>I1, E0, A0, S0	(0)	Week 7	ND
**G 2**	D5	(7)<br>I2, E2, A2, S1<br>Two lesions on the trunk.	(6)<br>I2, E1, A2, S1<br>Two lesions on the trunk.	(6)<br>I2, E1, A2, S1<br>Two lesions on the trunk.	(4)<br>I2, E0, A2, S0<br>Two lesions on the trunk.	(3)<br>I2, E0, A1, S0<br>Two lesions on the trunk.	(1)<br>I1, E0, A1, S0<br>One lesion on the trunk.	(0)		Week 6	ND
	D6	(8)<br>I3, E2, A2, S1<br>Three lesions on the trunk and four lesions on the abdomen.	(7)<br>I3, E1, A2, S1<br>Three lesions on the trunk and four lesions on the abdomen.	(6)<br>I3, E0, A2, S1<br>Three lesions on the trunk and four lesions on the abdomen.	(5)<br>I3, E0, A2, S0<br>Three lesions on the trunk and four lesions on the abdomen.	(3)<br>I2, E0, A1, S0<br>Four lesions on the abdomen.	(1)<br>I1, E0, A0, S0<br>One lesion on the abdomen.	(0)		Week 6	ND

	D7	(7)<br>I2, E2, A2, S1<br>Two lesions on the trunk.	(6)<br>I2, E1, A2, S1<br>Two lesions on the trunk.	(5)<br>I2, E0, A2, S1<br>Two lesions on the trunk.	(4)<br>I2, E0, A2, S0<br>Two lesions on the trunk.	(2)<br>I1, E0, A1, S0<br>One lesion on the trunk.	(1)<br>I1, E0, A0, S0<br>One lesion on the trunk.	(0)		Week 6	ND
	D8	(8)<br>I1, E2, A3, S2<br>One lesion on the thigh.	(7)<br>I1, E1, A3, S2	(6)<br>I1, E0, A3, S2	(3)<br>I1, E0, A2, S0	(2)<br>I 1, E0, A1, S0	(0)			Week 5	ND
**G 3**	D9	(7)<br>I2, E 1, A 3, S1<br>Seven lesions on the head	(5)<br>I2, S1, A2<br>Seven lesions	(4)<br>I2, A 2<br>Five lesions	(2)<br>I1, A 1<br>Two lesions	(1)<br>I1, A 0<br>One lesion	(0)			Week 5	ND
	D10	(6)<br>-I1, E 2, A 2, S2<br>-I1, E 1, A 1, S2<br>One lesion in both the head and trunk.	(4)<br>-I1, E 1, A 2, S1<br>-I1, E 0, A 1, S1	(3)<br>-I1, E 0, A 2, S1<br>-I1, E 0, A 1, S0	(2)<br>-I1, E 0, A 1, S0<br>-I1, E 0, A 1, S0	(1)<br>-I1, E 0, A 0, S0<br>-I1, E 0, A 0, S0	(0)			Week 5	ND
	D11	(8)<br>I2, E 1, A 3, S2. Eight lesions on the head.	(6)<br>I2, E 0, A 3, S1<br>Seven lesions	(5)<br>I2, S1, A2<br>Five lesions	(3)<br>I2, S0, A1<br>Three lesions	(2)<br>I2<br>S0, A0, E0<br>Two lesions	(1)<br>I1, S0, A0, E0<br>One lesion.	(0)		Week 6	ND
	D12	(7)<br>I1, A 2, E 2, S2<br>One lesion on the head.	(5)<br>I1, A 2, E 1, S1	(3)<br>I1, A 1, E1, S0	(2)<br>I1, A 1, E0, S0	(1)<br>I1, S0, A0, E0	(0)			Week 5	ND
**G 4**	D13	(7)<br>I1, A 2, E 1, S3<br>One lesion on the head.	(6)<br>I1, A 2, E 1, S2	(4)<br>I1, A 2, E0, S1	(2)<br>I1, A 1, E0, S0	(0)				Week 4	ND
	D14	(6)<br>I3, A 2, E0, S1<br>One lesion on the trunk, fore limb, and hind limb.	(5)<br>I3, A 2, E 0, S0.<br>One lesion on the trunk, fore limb, and hind limb.	(3)<br>I3, A0, E 0, S0<br>One lesion on the trunk, fore limb, and hind limb.	(1)<br>I1, A0, E 0, S0<br>One lesion on the trunk.	(0)				Week 4	ND

	D15	(8)<br>I2, A 2, E2, S2<br>Eight lesions on the trunk.	(6)<br>I 2, A 2, E1, S1	(4)<br>I 2, A2, E0, S0	(2)<br>I 2, A0, E0, S0	(1)<br>I 1, A0, E0, S0	(0)			Week 5	ND
	D16	(7.5)<br>-I 2, A 2, E 3, S0<br>-I2, A 2, E 3, S1<br>Two lesions on the rump.	(5)<br>-I 2, A 1, E1, S0<br>-I2, A 2, E 2, S0	(3)<br>-I 2, A 1, E0, S0<br>-I2, A 1, E 0, S0	(2)<br>-I 2, A 0, E0, S0<br>-I2, A 0, E 0, S0	(1)<br>-I 1, A 0, E0, S0<br>-I 0, A 0, E 0, S0	(0)			Week 5	ND
**G 5**	D17	(5)<br>A2, E1, S2<br>One lesion on the trunk.	(6)<br>A2, E1, I1, S2	(7)<br>A3, E0, I2, S2	(7)<br>A3, E0, I2, S2	(6)<br>A3, E0, I2, S1	(5)<br>A2, E0, I2, S1	(4)<br>A2, E0, I1, S1	(3)<br>A2, E0, I1, S0	No recovery was observed during the seven weeks period.	Untreated control group
	D18	(6)<br>A3, E3, S2<br>One lesion on the limb.	(7)<br>A3, E2, S2	(8)<br>A3, E2, S3	(7)<br>A3, E1, S3	(6)<br>A2, I1, S3	(5)<br>A2, I1, S2	(4)<br>A2, I1, S1	(4)<br>A2, I1, S1		
	D19	(8)<br>A3, E2, I 1, S2<br>Five lesions on the abdomen.	(9)<br>A3, E2, I 1, S3	(9)<br>A3, E2, I 1, S3	(8)<br>A3, E1, I 1, S3	(6)<br>A2, E0, I 1, S3	(5)<br>A2, E0, I 1, S2	(4)<br>A2, E0, I 0, S2	(3)<br>A2, E0, I 0, S1		
	D20	(7)<br>A2, E1, I 2, S2<br>One lesion on the head.	(8)<br>A3, E1, I 2, S2	(9)<br>A3, E1, I 2, S3	(8)<br>A3, E0, I 2, S3	(7)<br>A3, E0, I 2, S2	(6)<br>A2, E0, I 2, S2	(5)<br>A2, E0, I 2, S1	(3)<br>A1, E0, I 1, S1		

Lesion scores; A: alopecia, E: erythema, S: scales and crusts, I: induration. Lesion score was calculated according to Ural and Ultas (2008):

$$\text{Region Score} = \frac{\text{sum of the scores assigned to all lesions in the area}}{\text{number of lesions in this area}}$$

The numbers presented within parentheses represent the total score for the examined region. No adverse effects were detected in the treated cases. G1: animals were topically treated with clotrimazole, G2 was topically treated with clotrimazole and 1% itraconazole oral suspension, G3 received clotrimazole, itraconazole, and supportive treatment with multivitamins and minerals, and G4 received clotrimazole, itraconazole, supportive treatment, and vacderm vaccine. Animals in G5 were infected untreated.

Ural, K.; Ulutas, B. Immunization with *Trichophyton verrucosum* vaccine in hunter/jumper and dressage horses with naturally occurring *Trichophyton equinum* infection: A prospective, randomized, double blinded, placebo-controlled clinical trial. *J. Equine Vet. Sci.* **2008**, *28*, 590-593.

Supplementary Table S5. Clinical scoring and outcome of treatment regimens for treatment of cats naturally infected with *Microsporum canis*

Group	Cat	Clinical scoring									Clinical outcome	
		Week 0	Week 1	Week 2	Week 3	Week 4	Week 5	Week 6	Week 7	Week 8	Recovery	Relapse
**G 1**	C1	(6)<br>A2, E1, I 0, S3<br><br>One lesion on the paw	(5)<br>A2, E1, I0, S2,	(4)<br>A2, E2, I0, S0	(3)<br>A2, E1, I0, S0,	(3)<br>A2, E1, I0, S0	(2)<br>A2, E0, I0, S0,	(1)<br>A1, E0, I0, S0.	(1)<br>A1, E0, I0, S0.	(0)	Week 8	ND
	C2	(6)<br>A3, E0, I 1, S2	(6)<br>A3, E1, I 0, S2	(5)<br>A3, E1, I 1, S1	(5)<br>A3, E1, I 1, S1	(4)<br>A2, E1, I 0, S1	(2)<br>A1, E0, I 0, S1	(1)<br>A1, E0, I 0, S0	(0)		Week 7	ND
	C3	(7)<br>A3, E2, I1, S1<br>Two lesions on the trunk	(7)<br>A3, E2, I1, S1	(6)<br>A3, E2, I1, S1	(4)<br>A2, E0, I1, S1	(3)<br>A2, E0, I1, S0	(4)<br>A3, E0, I1, S1	(3)<br>A2, E0, I1, S0	(1)<br>A1, E0, I0, S0	(0)	Week 8	New lesions appear
	C4	(7)<br>A3, E2, I1, S2<br>One lesion on the scruff	(6)<br>A3, E0, I1, S2	(5)<br>A2, E0, I1, S2	(4)<br>A2, E0, I0, S2	(4)<br>A2, E0, I0, S2	(3)<br>A2, E0, I0, S1	(3)<br>A2, E0, I0, S1	(1)<br>A1, E0, I0, S0	(0)	Week 8	New lesions appear
**G 2**	C5	(5)<br>A2, E1, I0, S2<br>Lesion on the head	(5)<br>A2, E1, I0, S2	(4)<br>A1, E1, I0, S2	(3)<br>A1, E0, I0, S2	(2)<br>A1, E0, I0, S1	(1)<br>A1, E0, I0, S0	(0)			Week 6	ND
	C6	(7)<br>A2, E2, I2, S1<br>Lesions on the ears	(6)<br>A2, E1, I2, S1	(5)<br>A2, E0, I2, S1	(4)<br>A2, E0, I1, S1	(4)<br>A2, E0, I1, S1	(3)<br>A1, E0, I1, S1	(1)<br>A1, E0, I0, S0	(0)		Week 7	ND
	C7	(8)<br>A3, E2, I1, S2<br>One lesion on the head and one on the trunk	(7)<br>A2, E2, I1, S2	(6)<br>A2, E1, I1, S2	(5)<br>A2, E0, I1, S2	(3)<br>A2, E0, I0, S1	(2)<br>A1, E0, I0, S1	(1)<br>A1, E0, I0, S0	(0)		Week 7	ND

	C8	(7)<br>A2, E2, I0, S3<br>One lesion on the head	(6)<br>A2, E1, I0, S3	(5)<br>A2, E1, I0, S2	(4)<br>A2, E0, I0, S2	(3)<br>A2, E0, I0, S1	(2)<br>A1, E0, I0, S1	(1)<br>A1, E0, I0, S0	(0)		Week 7	ND
**G 3**	C9	(8)<br>A3, E2, I1, S2	(7)<br>A3, E1, I1, S2	(5)<br>A2, E0, I1, S2	(3)<br>A1, E0, I1, S1	(1)<br>A1, E0, I0, S0	(0)				Week 5	ND
	C10	(7)<br>A2, E2, I1, S2<br>One lesion on the neck	(6)<br>A2, E1, I1, S2	(5)<br>A2, E0, I1, S2	(3)<br>A1, E0, I1, S1	(2)<br>A1, E0, I1, S0	(1)<br>A1, E0, I0, S0	(0)			Week 6	ND
	C11	(8)<br>A2, E2, I1, S3	(7)<br>A2, E1, I1, S3	(6)<br>A2, E0, I1, S3	(5)<br>A2, E0, I1, S2	(3)<br>A2, E0, I0, S1	(1)<br>A1, E0, I0, S0	(0)			Week 6	ND
	C12	(7)<br>A2, E2, I0, S3<br>One lesion on the rump	(6)<br>A2, E1, I0, S3	(4)<br>A2, E0, I0, S2	(3)<br>A2, E0, I0, S1	(1)<br>A1, E0, I0, S0	(0)				Week 5	ND
**G 4**	C13	(9)<br>A3, E2, I3, S1<br>One lesion on the neck	(7)<br>A2, E1, I3, S1	(5)<br>A2, E1<br>I2, S0	(3)<br>A1, E0<br>I2, S0	(1)<br>A0, E0, I 1, S0	(0)				Week 5	ND
	C14	(6)<br>A3, E1, I1, S1<br>One lesion on the head.	(5)<br>A3, E0, I1, S1	(3)<br>A2, E0, I1, S0	(1)<br>A1, E0, I0, S0	(0)					Week 4	ND
	C15	(8)<br>A2, E2, I3, S1<br>One lesion on the neck	(7)<br>A2, E1, I3, S1	(5)<br>A2, E0, I2, S1	(3)<br>A1, E0, I2, S0	(1)<br>A0, E0, I1, S0	(0)				Week 5	ND
	C16	(7)<br>A2, E2, S2, I1<br>One lesion on the	(5)<br>A2, E 1, I 1, S1	(3)<br>A1, E0, I1, S1	(1)<br>A1, E0, I 0, S0	(0)					Week 4	ND

		back										
**G 5**	C17	(5)<br>A2, E2, I0, S1<br>One lesion on the scruff	(6)<br>A2, E2, I0, S2	(6)<br>A2, E1, I1, S2	(7)<br>A3, E0, I2, S2	(7)<br>A3, E0, I1, S3	(6)<br>A2, E0, I1, S3	(5)<br>A2, E0, I1,2S	(4)<br>A2, E0, I0, S2	(2)<br>A1, E0, I0, S1	No recovery was observed during the eight weeks period.	Untreated control group
	C18	(6)<br>A3, E2, I0, S1<br>One lesion on the trunk	(7)<br>A3, E3, I0, S1	(7)<br>A3, E2, I1, S1	(8)<br>A3, E1, I2, S2	(7)<br>A2, E1, I2, S2	(6)<br>A2, E0, I2, S2	(5)<br>A2, E0, I2, S1	(3)<br>A2, E0, I0, S1	(2)<br>A2, E0, I0, S0		
	C19	(8)<br>A2, E2, I2, S2<br>Four lesions on the back	(9)<br>A3, E2, I2, S2<br>Six lesions on the back	(8)<br>A3, E1, I2, S2<br>Seven lesions on the back	(7)<br>A3, E1, I1, S2<br>Seven lesions on the back	(6)<br>A3, E0, I1, S2<br>Seven lesions on the back	(5)<br>A3, E0, I1, S1<br>Seven lesions on the back	(3)<br>A2, E0, I0, S1<br>Seven lesions on the back	(2)<br>A2, E0, I0, S0<br>Seven lesions on the back	(1)<br>A1, E0, I0, S0<br>Seven lesions on the back		
	C20	(7)<br>A2, E2, I2, S1<br>One lesion on the neck	(8)<br>A3, E2, I2, S1	(6)<br>A3, E1, I2, S1	(5)<br>A3, E0, I1, S1	(4)<br>A2, E0, I2, S1	(3)<br>A2, E0, I0, S1	(3)<br>A2, E0, I0, S1	(2)<br>A2, E0, I0, S0	(1)<br>A1, E0, I0, S0		

Lesion scores; A: alopecia, E: erythema, S: scales and crusts, I: induration. Lesion score was calculated according to Ural and Ultas (2008):

$$\text{Region Score} = \frac{\text{sum of the scores assigned to all lesions in the area}}{\text{number of lesions in this area}}$$

The numbers presented within parentheses represent the total score for the examined region. No adverse effects were detected in the treated cases. G1: animals were topically treated with clotrimazole, G2 was topically treated with clotrimazole and 1% itraconazole oral suspension, G3 received clotrimazole, itraconazole, and supportive treatment with multivitamins and minerals, and G4 received clotrimazole, itraconazole, supportive treatment, and vacderm vaccine. Animals in G5 were infected untreated.

Ural, K.; Ulutas, B. Immunization with *Trichophyton verrucosum* vaccine in hunter/jumper and dressage horses with naturally occurring *Trichophyton equinum* infection: A prospective, randomized, double blinded, placebo-controlled clinical trial. *J. Equine Vet. Sci.* **2008**, *28*, 590-593.

Supplementary Table S6. Mean score of *Microsporum canis*-infected and treated dogs in different treatment groups

Groups	Week 0	Week 1	Week 2	Week 3	Week 4	Week 5	Week 6	Week 7
G1	7.5±0.65, (5.45-9.55)	7.25±0.25, (4.86-9.64) [a]	6.25±0.48, (4.73-7.77) [b]	5.25±0.48, (3.73-6.77) [b]	4±0.58, (2.16-5.84) [b]	2±0.41, (0.7-3.3) [b]	0.75±0.25, (-0.05-1.55) [b]	0[b]
G2	7.5±0.29, (6.58-8.42)	6.5±0.29, (5.58-7.42) [ab]	5.75±0.25, (4.95-6.55) [b]	4±0.41, (2.7-5.3) [b]	2.5±0.29, (1.58-3.42) [bc]	0.75±0.25, (-0.05-1.55) [c]	0 [c]	0[b]
G3	7±0.41, (5.7-8.3)	5±0.41, (3.7-6.3) [b]	3.75±0.48, (2.23-5.27) [c]	2.25±0.25, (1.45-3.05) [c]	1.25±0.25, (0.45-2.05) [cd]	0.25±0.25, (-0.55-1.05) [c]	0[c]	0[b]
G4	7±0.41, (5.7-8.3)	5.5±0.29, (4.58-6.42) [ab]	3.5±0.29, (2.58-4.42) [c]	1.75±0.25, (0.95-2.55) [c]	0.5±0.29, (-0.42-1.42) [d]	0[c]	0[c]	0[b]
G5	6.5±0.65, (4.45-8.55)	7.5±0.65, (5.45-9.55) [a]	8.25±0.48, (6.73-9.77) [a]	7.5±0.25, (6.58-8.42) [a]	6.25±0.25, (5.45-7.05) [a]	5.25±0.25, (4.45-6.05) [a]	4.25±0.25, (3.45-5.05) [a]	3.25±0.25, (2.45-4.05) [a]
p-value	0.604	0.014	<0.0001	<0.0001	<0.0001	<0.0001	<0.0001	<0.0001

Results are expressed as mean± SEM (standard error of the mean), 95% CI. [a-d] Means with various superscript letters within the same column indicate significant difference at $p<0.05$. G1: animals were topically treated with clotrimazole, G2 was topically treated with clotrimazole and 1% itraconazole oral suspension, G3 received clotrimazole, itraconazole, and supportive treatment with multivitamins and minerals, and G4 received clotrimazole, itraconazole, supportive treatment, and Vacderm vaccine. Animals in G5 were infected untreated.

Supplementary Table S7. Mean score of *Microsporum canis*-infected and treated cats in different treatment groups

Groups	Week 0	Week 1	Week 2	Week 3	Week 4	Week 5	Week 6	Week 7	Week 8
G1	6.5±0.29, (5.58-7.42)	6±0.41, (4.7-7.3)	5±0.41, (3.7-6.3) [ab]	4±0.41, (2.7-5.3) [b]	3.5±0.29, (2.58-4.42) [b]	2.75±0.48, (1.23-4.27) [b]	2±0.58, (0.16-3.84) [b]	0.75±0.25, (-0.05-1.55) [b]	0[b]
G2	6.75±0.63, (4.75-8.75)	6±0.41, (4.7-7.3)	5±0.41, (3.7-6.3) [ab]	4±0.41, (2.7-5.3) [b]	3±0.41, (1.7-4.3) [b]	2±0.41, (0.7-3.3) [bc]	0.75±0.25, (-0.05-1.55) [bc]	0[b]	0[b]
G3	7.5±0.29, (6.58-8.42)	6.5±0.29, (5.58-7.42)	5±0.41, (3.7-6.3) [ab]	3.5±0.5, (1.91-5.05) [b]	1.75±0.48, (0.23-3.27) [bc]	0.5±0.29, (-0.42-1.42) [cd]	0[c]	0[b]	0[b]
G4	7.5±0.65, (5.45-9.55)	6±0.58, (4.58-6.42)	4±0.58, (2.16-5.84) [b]	2±0.58, (0.16-3.84) [b]	0.5±0.29, (-0.42-1.42) [c]	0[d]	0[c]	0[b]	0[b]
G5	6.5±0.65, (4.45-8.55)	7.5±0.65, (5.45-9.55)	6.75±0.48, (5.23-8.27) [a]	6.75±0.63, (4.75-8.75) [a]	6±0.71, (3.75-8.28) [a]	5±0.71, (2.75-7.25) [a]	4±0.58, (2.16-5.84) [a]	2.75±0.48, (1.23-4.27) [a]	1.5±0.29, (0.58-2.42) [a]
p-value	0.468	0.177	0.012	<0.0001	<0.0001	<0.0001	<0.0001	<0.0001	<0.0001

Results are expressed as mean± SEM (standard error of the mean), 95% CI. [a-d] Means with various superscript letters within the same column indicate significant difference at $p<0.05$. G1: animals were topically treated with clotrimazole, G2 was topically treated with clotrimazole and 1% itraconazole oral suspension, G3 received clotrimazole, itraconazole, and supportive treatment with multivitamins and minerals, and G4 received clotrimazole, itraconazole, supportive treatment, and Vacderm vaccine. Animals in G5 were infected untreated.

Supplementary Figure S1. *T. indotineae* causing ringworm in an eight-month-old local breed dog. Crusting and scaling lesions on the lumbosacral area (A) and on head (B). Direct microscopic examination of hair sample revealed the presence of ectothrix spores (C). Flat granular colonies slightly raised in the center "Button-shape" with beige surface and light brown reverse colour on SDA+C+C (D and E). LPCB mount revealed clusters of numerous spherical with occasional pyriform shape microconidia, spiral hyphae, and clavate-shape macroconidia.

Supplementary Figure S2. Agarose gel electrophoresis for the amplified products of ITS region of fungal isolates. (A) M: 100 bp molecular size marker; lanes 1-9 and 11-19: *Microsporum canis* at 720 bp; lanes: 10,21: *Trichophyton mentagrophytes* at 680 bp; lane 20: *Microsporum gypseum* at 650 bp. (B) M: 100 bp molecular size marker, lanes 22,39,43 *Microsporum canis* at 720 bp; lanes: 24-26, 28, 29, 31,32,35 and 42: *Trichophyton mentagrophytes* at 680 bp, lanes 23,27,30 and 33: *Trichophyton verrucosum* at 678 bp, lanes 37,38,44: *Malassezia pachydermatis* at 750 bp, and lanes 40 and 41: *Trichosporon asahii* at 540 bp.

Supplementary Figure S3. *Mva1* RFLP patterns of dermatophyte species and yeasts ITS amplicons. (A) Lane M, 50 -bp molecular size marker; lanes 1-9 and 11-19: *Microsporum canis* at 441, 156, 103, and 28 bp; lanes 10,21: *Trichophyton mentagrophytes* at 405, 124, 90, and 53 bp; lane 20: *Microsporum gypseum* at 400 and 250 bp. (B) Lane M: 50 -bp molecular size marker, lanes 22, 39, 43: *M. canis* at 441, 156, 103, 28 bp; lanes 24-26, 28, 29, 31, 32, 35, and 42: *T. mentagrophytes* at 405, 124, 90, 53 bp; lanes 23, 27, 30, and 33: *T. verrucosum* at 517, 141, and 20 bp; lanes 37, 38, 44*: Malassezia pachydermatis* at 380, 250, 120 bp and lanes 40 and 41: *Trichosporon asahii* at 210 and 330 bp.

Supplementary Figure S4. Ectoparasites detected in the examined animals. A: *Rhipicephalus sanguineous* tick (100 X), B: *Ctenocephalides spp.* flea (100 X), C: *Heterodoxus spiniger* adult louse (100 X), D: *Trichodectes canis* adult louse (100 X), E: *Otodectes cynotis* adult mite (100 X), F: arrows refer to *Demodex canis* egg and adult mite (400 X), and G: *Sarcoptes scabei* adult mite (100 X).

Supplementary Figure S5. Treatment of a Pit Bull dog with clotrimazole twice daily for 2 weeks. A: an area of alopecia and scales on tail. B: 3 weeks post treatment. C: 7 weeks post treatment.

Supplementary Figure S6. Treatment of a 1-year-old Persian female cat with clotrimazole. A. a circular area of scaling, alopecia, and erythema on dorsal aspect of the right front paw, B: 2 weeks post treatment, and C: 6 weeks post treatment.

Supplementary Figure S7. Treatment of a Golden Retriever dog with clotrimazole and itraconazole. A: Alopecia, scaling and erythema on the rump of the dog, B. 1 week post treatment, C: 2 weeks post treatment, and D: 4 weeks post treatment.

Supplementary Figure S8. Treatment of a 7-year-old Persian male cat with clotrimazole and itraconazole. A: a circular area of alopecia and erythema on face, B: 1 week post treatment, C: 2 weeks post treatment, D: 3 weeks post treatment, E: 4 weeks post treatment, and F: 5 weeks post treatment.

Supplementary Figure S9. Infected Yorkshire Terrier dog (a member of the control untreated group) A: a circular area of hair loss and erythema on the rump, B: follow-up after a week, C: after 4 weeks.
