# Supplementary figures and images for: Molecular Diagnostics and Control of Zoonotic Dermatophytosis: First Detection of Trichophyton indotineae in a Dog in Africa
Source: Animals (Basel). 2025 Sep 7;15(17):2622. doi: 10.3390/ani15172622 (PMC12427446; doi:10.3390/ani15172622)

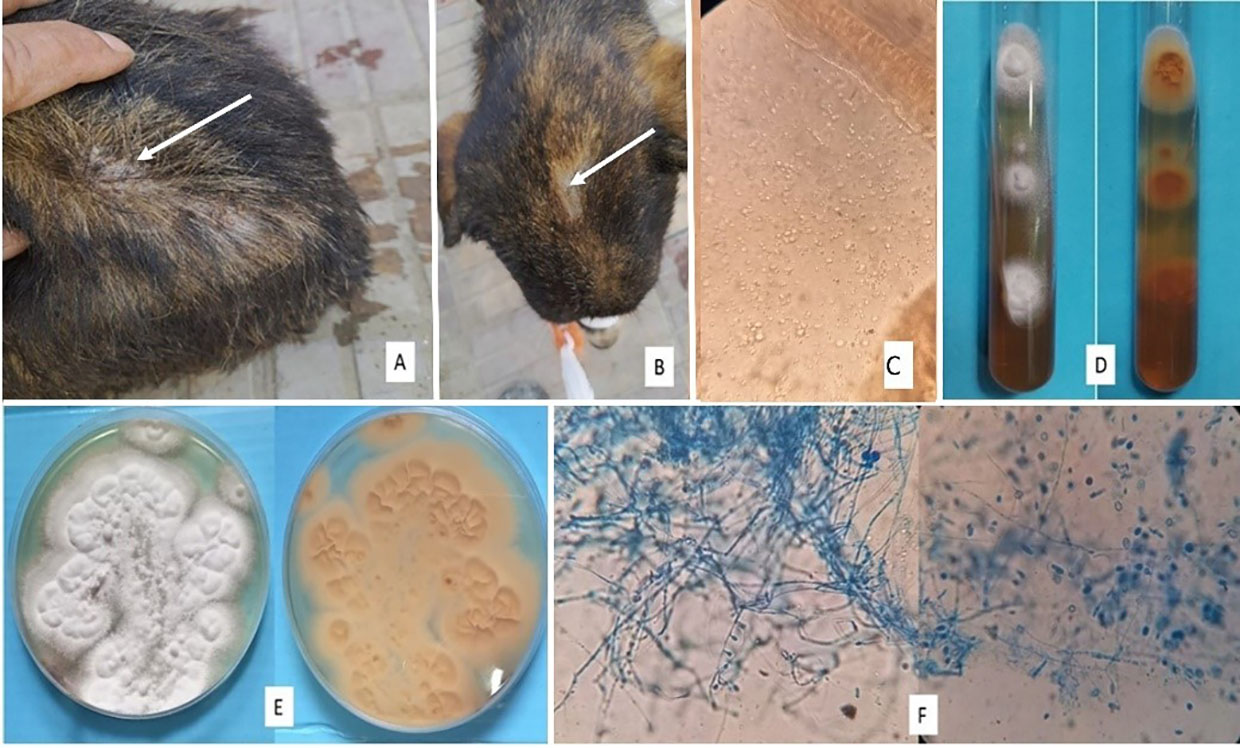

Supplement: Supplementary file 1 [file animals-15-02622-s001.zip › supplementary Figure S1.jpg]

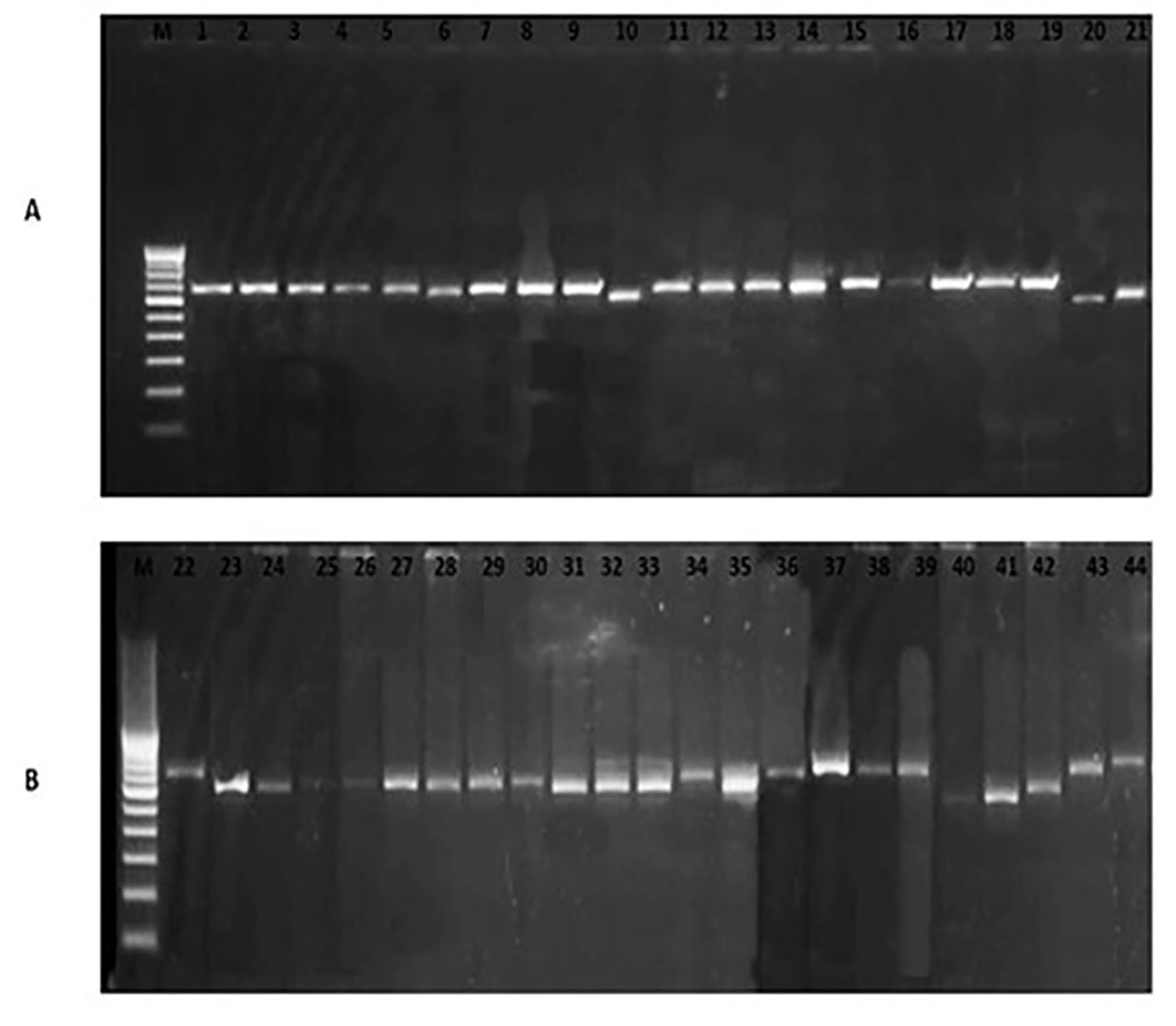

Supplement: Supplementary file 1 [file animals-15-02622-s001.zip › supplementary Figure S2.jpg]

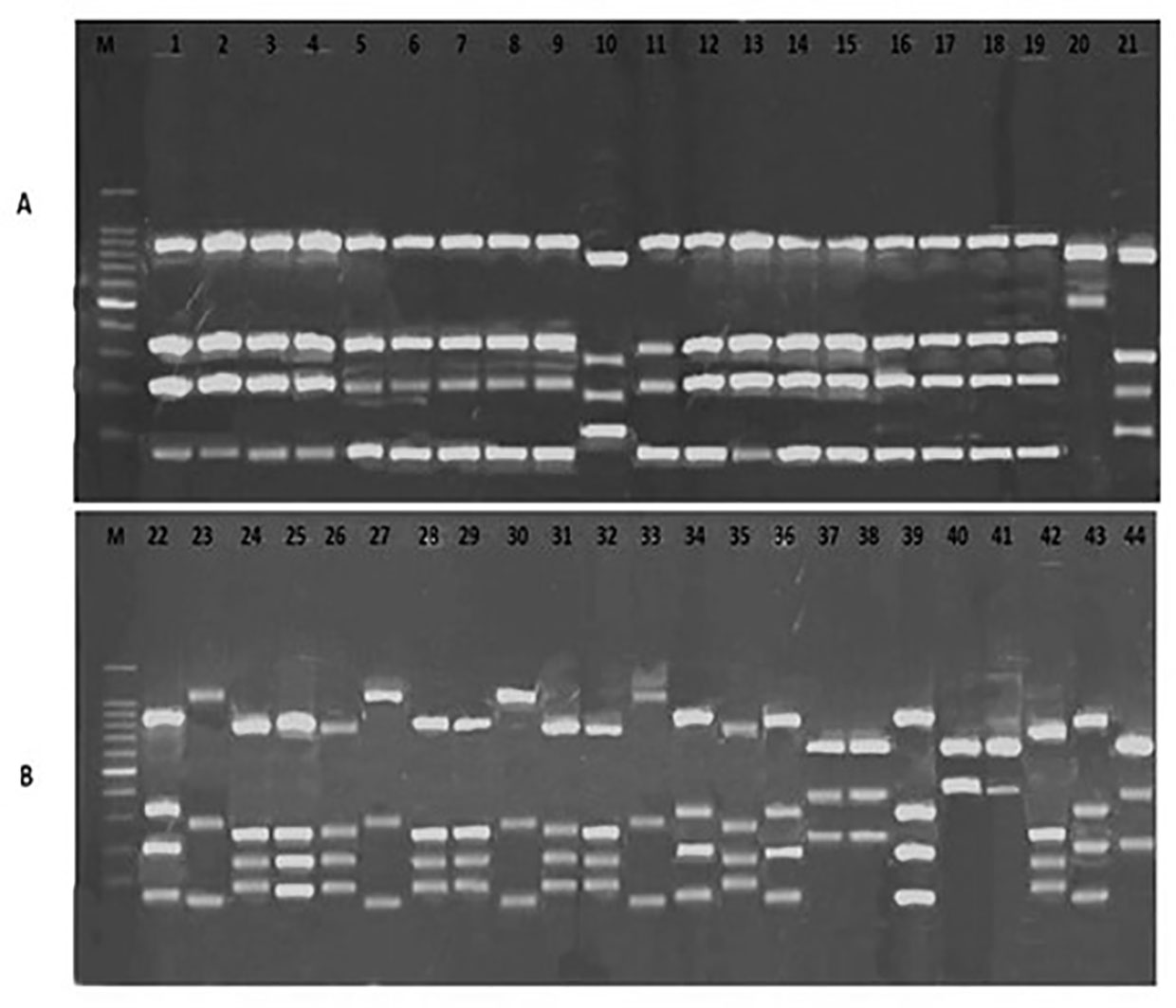

Supplement: Supplementary file 1 [file animals-15-02622-s001.zip › supplementary Figure S3.jpg]

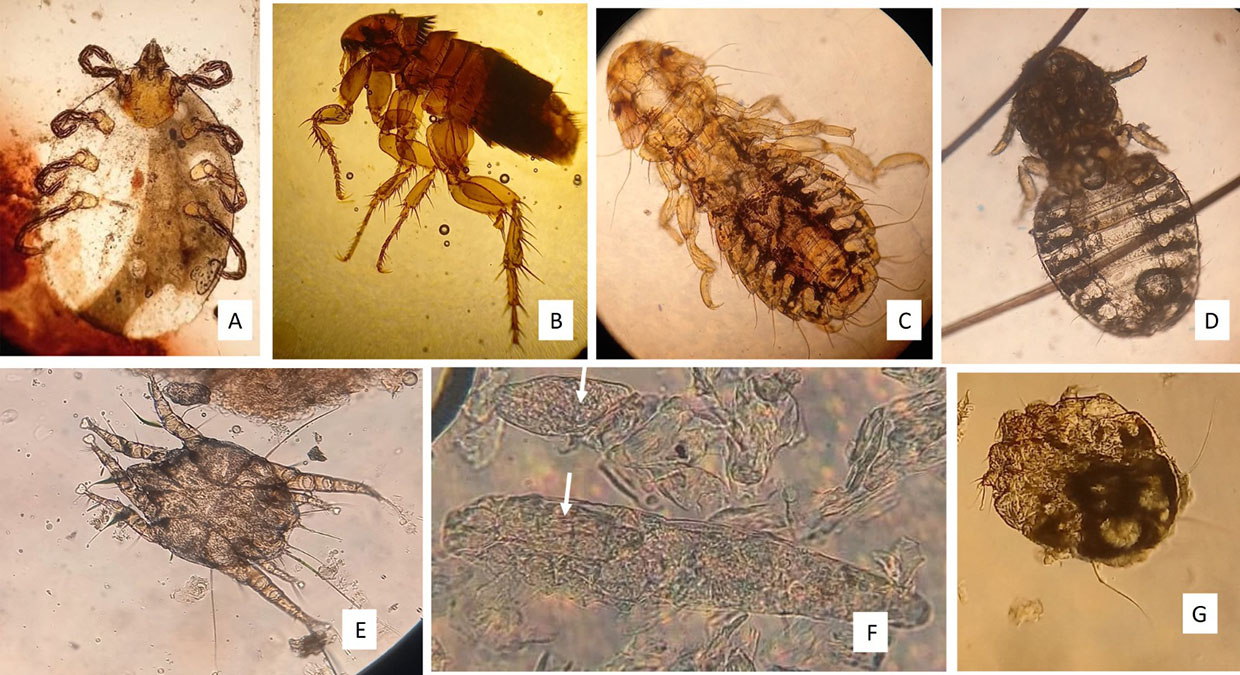

Supplement: Supplementary file 1 [file animals-15-02622-s001.zip › supplementary Figure S4.jpg]

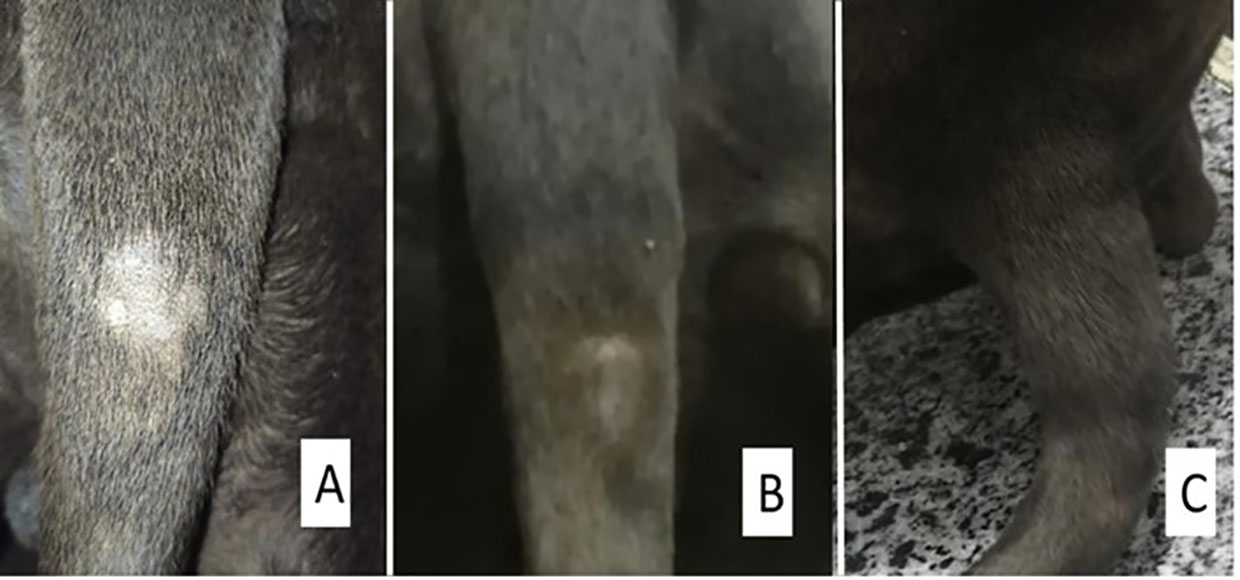

Supplement: Supplementary file 1 [file animals-15-02622-s001.zip › supplementary Figure S5.jpg]

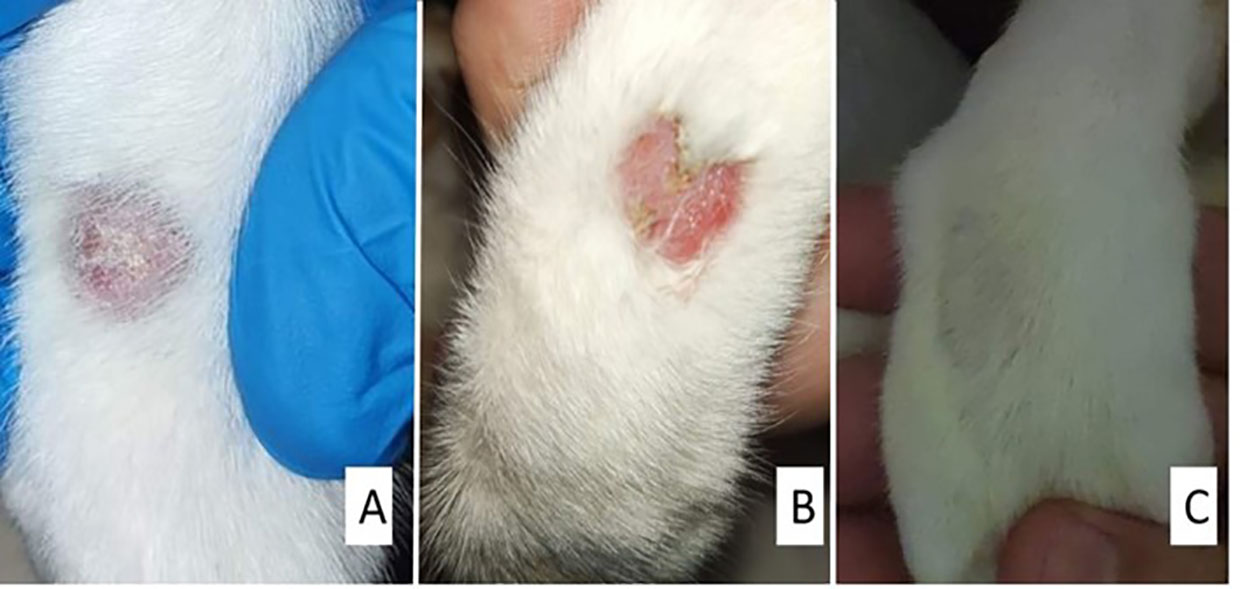

Supplement: Supplementary file 1 [file animals-15-02622-s001.zip › supplementary Figure S6.jpg]

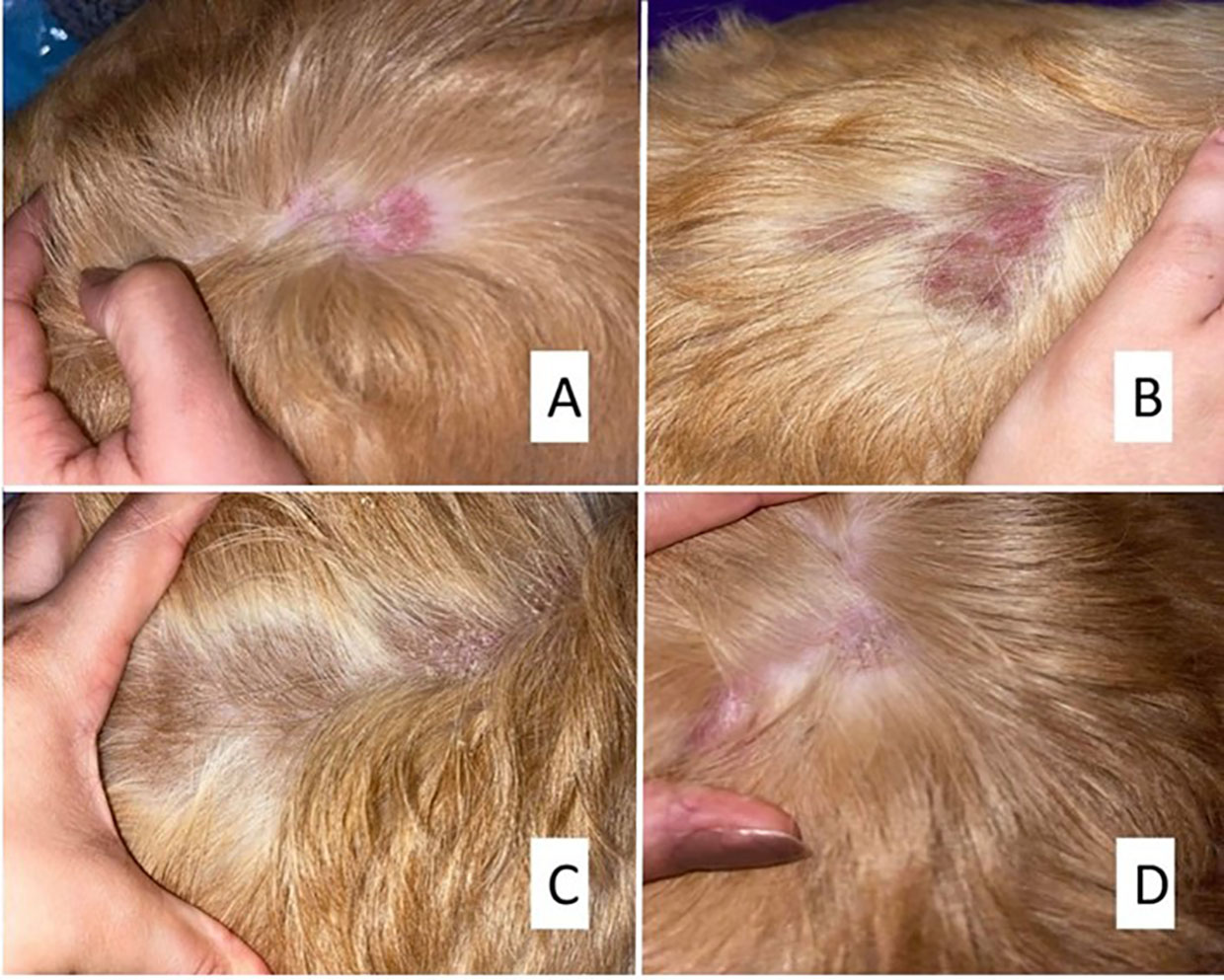

Supplement: Supplementary file 1 [file animals-15-02622-s001.zip › supplementary Figure S7.jpg]

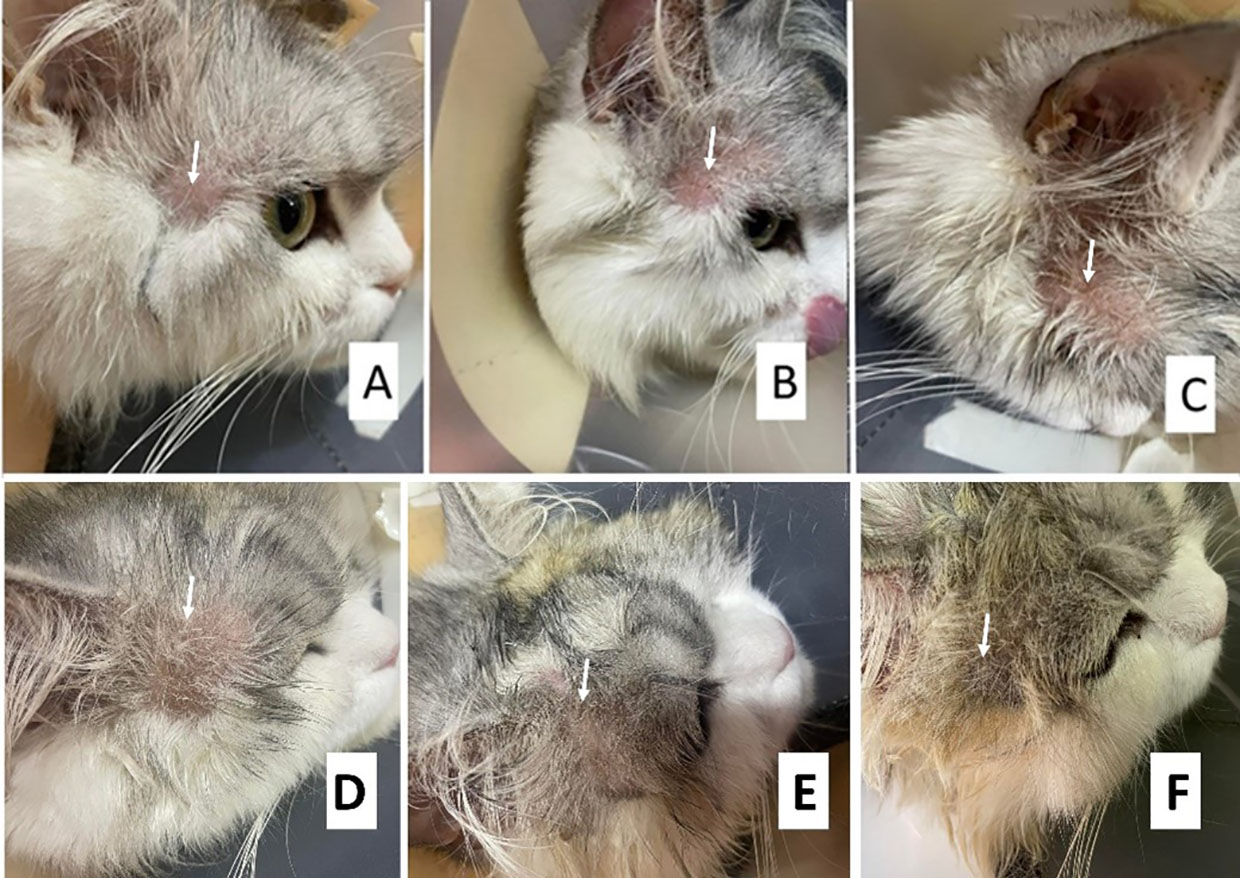

Supplement: Supplementary file 1 [file animals-15-02622-s001.zip › supplementary Figure S8.jpg]

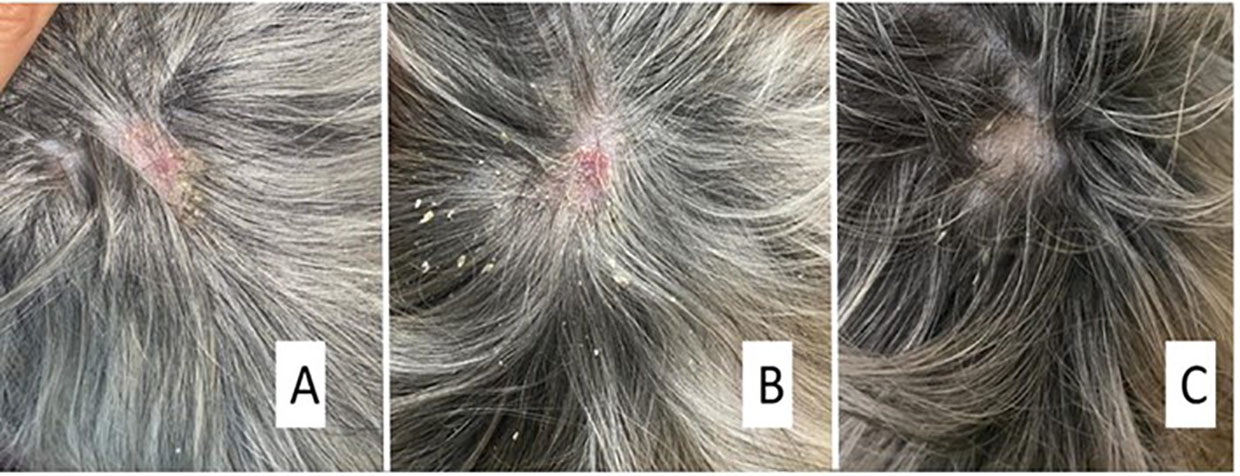

Supplement: Supplementary file 1 [file animals-15-02622-s001.zip › supplementary Figure S9.jpg]
